# Overcoming the fragility – X-ray computed micro-tomography elucidates brachiopod endoskeletons

**DOI:** 10.1186/s12983-014-0065-x

**Published:** 2014-09-27

**Authors:** Ronald Seidel, Carsten Lüter

**Affiliations:** Current address: Max Planck Institute of Colloids and Interfaces, Potsdam-Golm Science Park, Am Mühlenberg 1 OT Golm, 14476 Potsdam, Germany; Museum für Naturkunde, Leibniz Institute for Evolution and Biodiversity Science, Invalidenstrasse 43, 10115 Berlin, Germany

**Keywords:** Brachiopoda, Endoskeleton, Spicules, X-ray, Micro-computed tomography (μCT), Interactive 3D model, *Rectocalathis schemmgregoryi* n. gen., n. sp.

## Abstract

**Introduction:**

The calcareous shells of brachiopods offer a wealth of informative characters for taxonomic and phylogenetic investigations. In particular scanning electron microscopy (SEM) has been used for decades to visualise internal structures of the shell. However, to produce informative SEM data, brachiopod shells need to be opened after chemical removal of the soft tissue. This preparation occasionally damages the shell. Additionally, skeletal elements of taxonomic/systematic interest such as calcareous spicules which are loosely embedded in the lophophore and mantle connective tissue become disintegrated during the preparation process.

**Results:**

Using a nondestructive micro-computed tomography (μCT) approach, the entire fragile endoskeleton of brachiopods is documented for the first time. New insights on the structure and position of tissue-bound skeletal elements (spicules) are given as add ons to existing descriptions of brachiopod shell anatomy, thereby enhancing the quality and quantity of informative characters needed for both taxonomic and phylogenetic studies. Here, we present five modern, articulated brachiopods (*Rectocalathis schemmgregoryi* n. gen., n. sp.*, Eucalathis* sp.*, Gryphus vitreus, Liothyrella neozelanica* and *Terebratulina retusa*) that were X-rayed using a Phoenix Nanotom XS 180 NF. We provide links to download 3D models of these species, and additional five species with spicules can be accessed in the Supplemental Material. In total, 17 brachiopod genera covering all modern articulated subgroups and 2 inarticulated genera were X-rayed for morphological analysis. *Rectocalathis schemmgregoryi* n. gen., n. sp. is fully described.

**Conclusion:**

Micro-CT is an excellent non-destructive tool for investigating calcified structures in the exo- and endoskeletons of brachiopods. With high quality images and interactive 3D models, this study provides a comprehensive description of the profound differences in shell anatomy, facilitates the detection of new delicate morphological characters of the endoskeleton and gives new insights into the body plan of modern brachiopods.

**Electronic supplementary material:**

The online version of this article (doi:10.1186/s12983-014-0065-x) contains supplementary material, which is available to authorized users.

## Introduction

**Figure 1:**
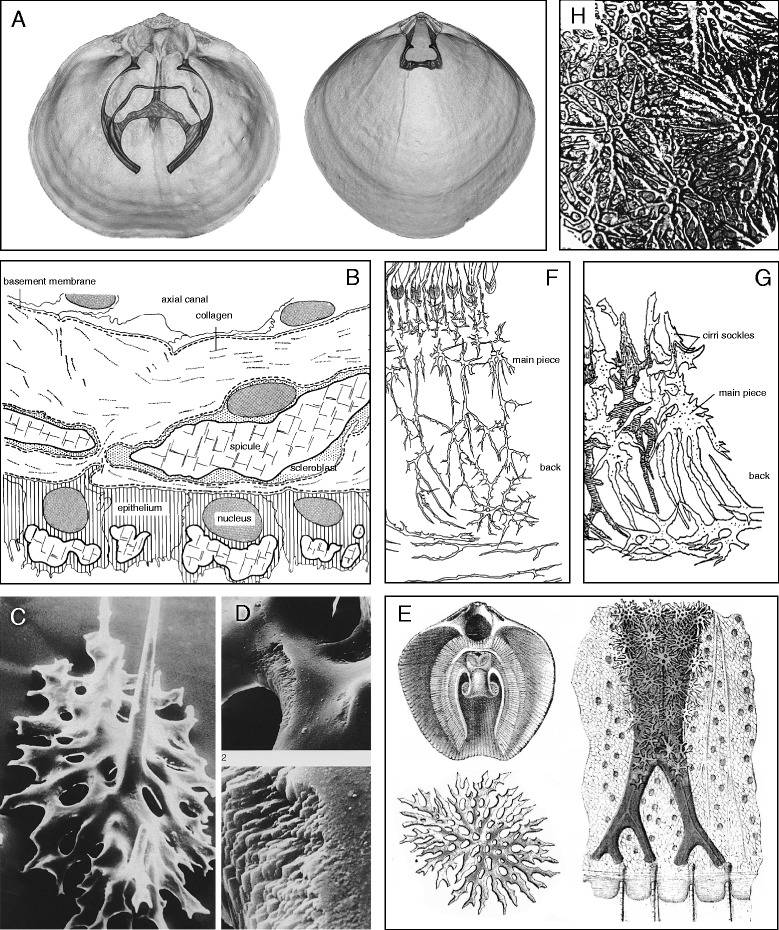
**Mineralized endo- and exoskeleton of brachiopods. A)** Interior of dorsal valves with “long loop” of *Calloria inconspicua* (Sowerby, 1846) (left) and “short loop” of *Liothyrella neozelanica* Thomson, 1918 (right) (R. Seidel). **B)** Stylised section showing the distribution of spicules in the epithelium and connective tissue of the lophophore of *Terebratulina retusa* (Linnaeus, 1758) according to Williams [4]. **C/D)** SEM of a spicule from the mantle of *T. retusa* with **(D)** the inner layer structure exposed in an area of recent biomineralisation [3]. **E)** The lophophoral endoskeleton and spicules in the mantle covering the pallial sinus of *T. retusa* by Eudes-Deslongchamps [7]. **F/G)** Spicules in the lophophore **F)** without and **G)** with cirri sockles at the bases of the tentacles [8]. **H)** Spicules of the dorsal body wall of *L. neozelanica* [12].

The morphology of brachiopod shells has been of interest to scientists for more than two centuries. In articulated brachiopods such as Rhynchonellida, Thecideida and Terebratulida the shell develops through calcite biomineralisation processes. Both dorsal and ventral valves usually consist of two (sometimes three) calcite layers comprising an outer, hard and protective nanocrystalline layer and a much thicker, inner secondary layer built from a hybrid organic/inorganic fibre composite material [1]. As outgrowth from this secondary shell layer of the dorsal valve [2], the brachidium forms a lophophore support [3] consisting of the crura and the loop-forming brachidia (Figure 1A). It is ensheathed in outer epithelium which controls growth by simultaneous secretion and resorption [4]. However, there are also calcareous structures within the soft tissue in many exclusively articulated brachiopods which are never associated with the two calcareous valves. Those structures are referred to as spicules and are defined as small irregular bodies of calcite secreted by scleroblasts within the connective tissue of the mantle and lophophore [4] (Figure 1B). The spicules are products of endodermal-mesenchymal cells [5] and are therefore a true endoskeleton. Inside a scleroblast, a spicule is separated from all other cell contents, presumably by a protein layer. The surface of a spicule is a rather smooth and thin calcite layer, but the actual body consists of highly declined laminae [4] (Figure 1C, D) which in turn are composed of calcitic spherules about 200 nm in diameter [6].

A true spicular endoskeleton is found only in articulated brachiopods where it is strongly developed in all species of the Terebratulidae, Platidiidae and Kraussinidae. In the families Megathyrididae, Dallinidae, Laqueidae and Thecideidae it is only developed in some species, and it does not occur at all in the Terebratellidae [4]. Spicules were first described in detail by Hancock [9] in *Terebratulina retusa* (Linnaeus, 1758), by Lacaze-Duthiers [10] in *Lacazella mediterranea* (Risso 1826) and extensively by Eudes-Deslongchamps [8] in *T. retusa* (Figure 1E), *Kraussina rubra* (Pallas, 1766), *Gryphus vitreus* (Born, 1778), *Megerlia truncata* (Linnaeus, 1767) and *Platidia anomioides* (Scacchi & Philippi in Philippi, 1844). Based on comprehensive investigations of the material obtained from the Valdivia- (1898-1899) and Gauß-Expeditions (1901-1903), F. Blochmann [8,11] tested the usefulness of the calcareous bodies for species diagnostics when other shell characters fail (Figure 1F, G). Despite existing illustrations of minute single spicules [12] (Figure 1H), the fragility of the endoskeleton limits the ability to visualise and therefore understand the anatomy of the endoskeleton as a whole. However, understanding the anatomy of the endoskeleton is essential for taxonomic and systematic analyses, as well as for any hypotheses on the functional properties of the endoskeleton of brachiopods.

Scanning electron microscopy (SEM) has been used for decades to visualise biological structures of interest in brachiopod shells, such as muscle scars, punctae, teeth, crura and the brachidium [4,13] and references therein. However, this method is limited by the fact that it is only surface-sensitive. Studying hard tissue of modern brachiopods with SEM requires treatment with chemical dissolvers to remove the soft tissue, which can lead to the damage of the shell. Additionally, endoskeletal elements such as calcareous spicules which are loosely embedded within the lophophore and mantle connective tissue disintegrate during the preparation process. Despite exhaustive research on hard tissue of brachiopods using high resolution SEM-imaging, a pictured overview of the occurrence, abundance and exact spatial distribution of endoskeletal spicules within the soft body has been lacking – until now.

In order to understand the inner shell morphology of either fossil and modern brachiopod shells X-ray radiographs of animals have been used since the 1970’s [14-16]. Hagadorn [17] was the first to use micro-computed tomography (μCT) and reconstructed 3D models to visualise the coiled lophophore support structures inside pyritized *steinkerns* of the Silica Shale brachiopod *Paraspirifer bownockeri* (Stewart, 1927). With increasing resolution and practicability of X-ray devices within the last decade computed tomography has become a valuable adjunct technique. A few studies have been carried out since on brachiopod shell morphology using micro-computed tomography ([18-22] as well as synchrotron radiation X − ray tomographic microscopy (SRXTM), which provides an even higher image fidelity [23,24].

The majority of analyses of fossil brachiopods using a microtomograph were carried out by Pakhnevich [18-21]. In 2010 he included 6 modern species in his investigations showing developmental series of the brachidium of *Macandrevia cranium* (Müller, 1776), the spiculation of the mantle connective tissue of the ventral valve of *T. retusa* and scans of *Eucalathis murrayi* (Davidson, 1878). Unfortunately, details of the scanning parameters and the scanning set up are missing (e.g. scans performed wet or dry, in ddH_2_O or EtOH). The presented images do not include all the information μCT scans actually are able to provide. For instance, data on spiculation within the lophophore and mantle *of T. retusa* or *E. murrayi* is limited, although micro computed tomography appears highly applicable for such tasks.

By using the non-invasive μCT approach, we were able to document the internal shell anatomy and to visualise the shells of modern brachiopods. We added structural and positional data on tissue-bound skeletal elements (e.g. spicules) to existing descriptions of brachiopod shell anatomy. We scanned long-term preserved specimens from the historical wet collection of the Museum für Naturkunde Berlin, Germany as well as recently collected samples which represent 19 brachiopod genera covering all articulated subgroups and two genera of (inarticulated) craniids. Here, we illustrate whole mount scans of five representative species to underline the capability of the non-invasive μCT approach for visualising hard tissue of brachiopods and present new characters of the endoskeleton with a SEM-comparable resolution of approximately 10 μm. Comprehensive descriptions of the spicule arrangements within the brachiopod soft tissue are given in the results chapter (for results on the remaining 5 species with spicules see Supplemental Material). One of the five species illustrated in detail was found during the deep-sea expedition SO 205 “Mangan” on manganese nodules in the Central East Pacific and is described as a new species *Rectocalathis schemmgregoryi* n. gen., n. sp.

## Results

Modern brachiopods exhibit manifold spiculation. The spicules vary in shape and spatial distribution within the soft tissue, covering almost the entire visceral cavity or only parts of it. The spicules are readily visible within reconstructed 2D image stacks and in rendered three-dimensional visualizations. Scan parameters have to be set specifically for each specimen due to shell differences in thickness and density. The spiculation varies within the tissue of single specimens (e.g. in *P. anomioides* and *Pajaudina atlantica* Logan, 1988). With respect to enhanced character visualisation, the valves and internal characters of all scanned species are coloured. Please note that the unified colouration of brachidia, crura and other lophophoral supportive structures such as median lamellae or peribrachial ridge refers to the functional properties of lophophoral support, not to homology. The terminology used in this study is in accordance with the terminology of the Treatise of Invertebrate Paleontology, Part H, Brachiopoda Revised, Vol. 1. Please use the Hand Tool in Adobe Acrobat to activate the 3D models and to use all intended functions (see tool bar > Enable virtual sectioning). For specimens used in this study see Table 1 and Additional file 1: Table S1. Please see Table 2 for volume analyses and morphometric measurements of the species shown in this paper and Additional file 2: Table S2 for volume analyses and morphometric measurements of all species of the initial study.

**Table 1:** **Species presented within this article**

**Species**	**Location**	**Date**	**GPS**	**Depth (m)**	**Collector**	**ZMB**
*T. retusa*	Norway	-	-	-	Sars	Bra 2253
*R. schemmgregoryi* n. gen., n. sp.	SO205/St. 40, NE Pacific	05.05.2010	011°47*'*56*''*N 116°49*'*85*''*W	3954	P. Martinez Arbizu	Bra 2254
*Eucalathis* sp.	RV MSM19/1076-1, S-Atlantic, SW of Africa	04.12.2011	040°22.46*'*S 014°53.77*'*E	2018	N. Furchheim	Bra 2351
*G. vitreus*	Naples, Italy, Mediterranean Sea	-	-	-	F. Blochmann	Bra 2261
*L. neozelanica*	Doubtful Sound, Bauza Island, NZ	12.06.1996	045°22*'*03*''*S 167°00*'*70*''*E	-	P. Meredith	Bra 2255

For the remaining species of the initial study (see Additional file 1: Table S1).

**Table 2:** **Volume analyses and morphometric measurements using VG Studio Max 2.1**

**Species**	**Vol. Total**	**Vol. DV**	**Vol. VV**	**Vol. Spic Loph.**	**Vol. Spic. DV**	**Vol. Spic VV**	**DV Length**	**VV Lenght**	**DV Width**	**VV Width**	**DV Heigth**	**VV Heigth**
*T. retusa*	194,2	88,4	104,5	1,8	1,1	1,23	16,4	18,2	14,7	14,7	3,6	5
*R. schemmgregoryi* n. gen., n. sp.	3,8	1,5	2	0,2	0,01	×	4	4,5	4,4	4,4	0,8	1
*Eucalathis* sp.	na	na	na	na	na	na	3,8	4,3	4	4	1,1	1,5
*G. vitreus*	682,2	223,3	441,2	5	7,1	1,7	29,3	32,6	28,5	28,5	7,6	10,8
*L. neozelanica*	1399	551	772,7	7,6	34	26	38,7	42,3	40	40	7,6	13

Volumes are in mm^3^ and length in mm. Abbr.: DV = dorsal valve, *na* = not applicable, VV = ventral valve, Spic Loph = spiculation within lophophore, S*pic* DV/VV spiculation within mantle of dorsal/ventral valve, Vol = Volume, Vol. total = Vol. VV + DV + Loph + Spic, × = no spiculation. For volume analyses and morphometric measurements of all species of the initial study see Additional file 2: Table S2.

### *Terebratulina retusa* (Cancellothyrididae) – ZMB Bra 2253

**Figure 2:**
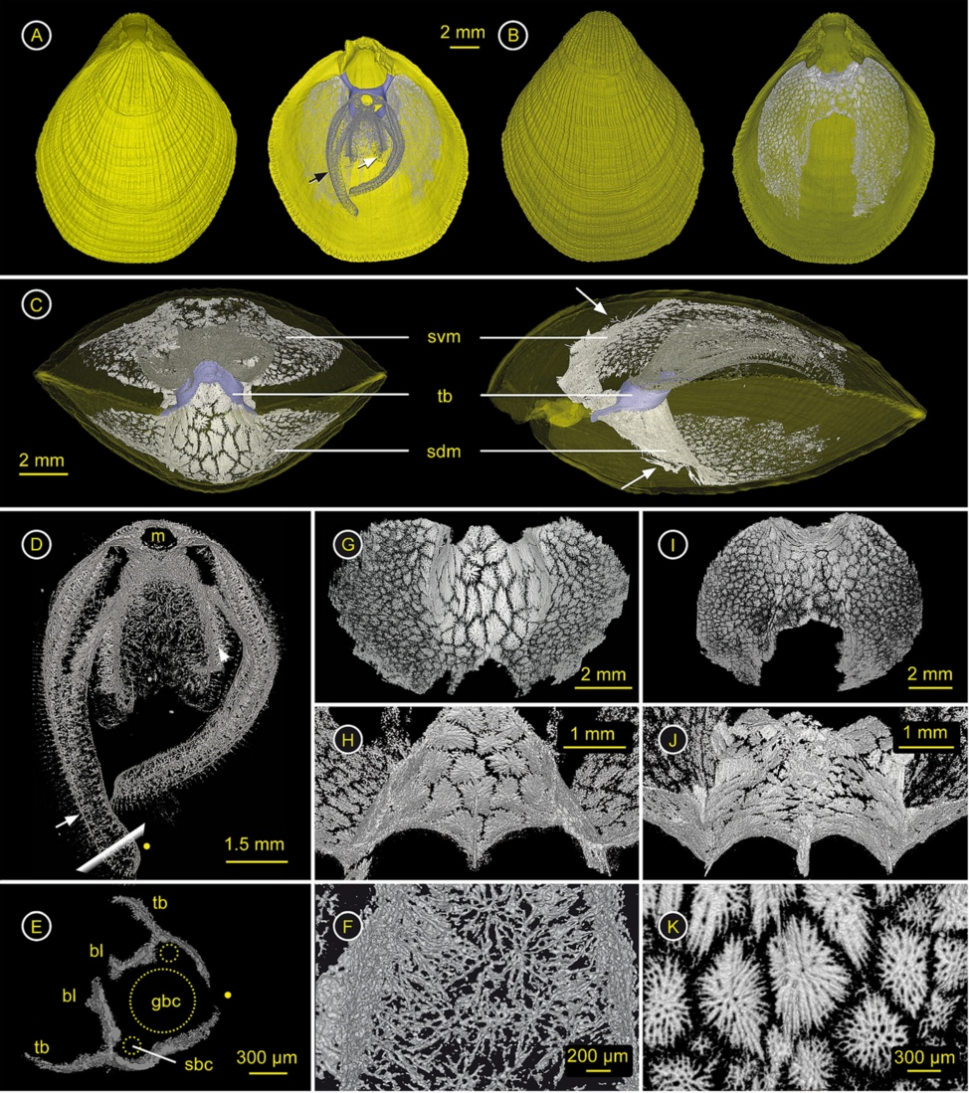
***Terebratulina retusa*** **(Cancellothyrididae) – ZMB Bra 2253.** Ventral valve = top & dorsal valve = bottom. Click **here** to download a 3D model of *T. retusa* (interactive PDF). **A)** left: Dorsal valve with ventral valve in the background; right: Interior of the dorsal valve with brachidium (purple), endoskeleton of the plectolophous lophophore (grey, black arrow = left lateral brachium, white arrow = right spiral brachium) and spiculation of the dorsal mantle. **B)** left: Ventral valve; right: Inside of ventral valve showing the incomplete, mesothyrid foramen and mantle spiculation. **C)** Anterior view through transparent shell with ventral valve on top, transverse band (tb) of the brachidium (purple) supports heavy spiculated lophophore (dark gray), ventral (svm) and dorsal mantle spiculation (sdm); right: Lateral view through transparent shell showing the short brachidium (purple), the endoskeleton of the ventrally ascending, plectolophous lophophore (dark gray), and the heavy mantle spiculation with excrescences on the posterior side into the visceral cavity (arrows). **D)** Ventral view of plectolophous lophophoral endoskeleton, left lateral brachium (arrow), right spiral brachium (arrow head), mouth (m). **E)** Cross section through lateral brachium (see **D)** for orientation), tentacle bases (tb), brachial lip (bl), and location of great (gbc) and small brachial channel (sbc). **F)** Spicules between the spiral brachia in front of the mouth. **G)** Anterior view of spicules within the dorsal body wall, smaller spicules (~0.6 mm in diameter) towards the shell margin and thick and large (~1.2 mm) star-shaped spicules in the center forming a posteriorly triple keeled skeleton, with **(H)** excrescences of single, elongated and branched spicules along the ridges, extending into the visceral cavity. **(I)** Anterior and posteroventrally (**J**) view of spiculation in the ventral mantle. **(K)** Magnification of star-shaped spicules from the median part of the dorsal mantle, max. size ~1.2 mm in diameter and max. 150 μm in thickness.

Spiculation - Spicules develop within the lophophore, and both the dorsal and ventral mantle (Figure 2A, Additional file 3). The spiculation throughout the soft tissue is strong. Within the plectolophous lophophore both lateral and spiral brachia are strongly spiculated at the bases of the tentacles, the brachial lip and the proximal part of the tentacles (Figure 2A, C, D). Between the posterior part of the spiral brachia the primarily isolated long and slender spicules are bigger and multibranched, building a network, which is most dense just in front of the mouth (Figure 2F). The brachial canals are almost completely surrounded by spicules (Figure 2E). The spiculation within the dorsal and ventral mantle is equally strong (Figure 2B, C), and consists of star-shaped spicules (Figure 2G, I, K). Towards the margin the size of the spicules decreases noticeably but the gaps between single spicules increase slightly, thus the entire mantle cavity is sheltered in an endoskeleton compartment. The rear of the spiculated body wall is triple keeled or ridged (Figure 2H, J). Along with the ridges, long and branched spicules reach into the visceral cavity (Figure 2C arrows).

### *Rectocalathis schemmgregoryi* n. gen., n. sp. (Chlidonophoridae) – ZMB Bra 2254

Systematic affiliation:

- Phylum BRACHIOPODA Duméril, 1806
- Subphylum RHYNCHONELLIFORMEA Williams & others, 1996
- Class RHYNCHONELLATA Williams & others, 1996
- Order TEREBRATULIDA Waagen, 1883
- Suborder TEREBRATULIDINA Waagen, 1883
- Superfamily CANCELLOTHYRIDOIDEA Thomson, 1926
- Family CHLIDONOPHORIDAE Muir-Wood, 1959
- Subfamily EUCALATHINAE Muir-Wood, 1965
- Genus *Rectocalathis* n. gen.

Type species - *Rectocalathis schemmgregoryi* n. sp.

Etymology - Genus named after its anatomically correct orientation (from Latin: *rectus* = upright, straight) when attached to its natural substrate due to the unusual position of the foramen.

Diagnosis - Small eucalathine brachiopod with a smooth to faintly costate shell and an extreme form of epithyrid (subumbonal) pedicle opening towards the ventral side of the animal.

Species *Rectocalathis schemmgregoryi* n. sp.

Type material - Holotype: ZMB Bra 2254, Paratypes: ZMB Bra 2350, 2352 (total: 3 specimens).

Type locality - NE Pacific, Clipperton Region, 11°47′56″N 116°49′85″W, Cruise SO 205 MANGAN, Station EBS 40, depth: 3954 m, May 5^th^ 2010.

Etymology - Species named after the late brachiopod researcher Mena-Daniela Schemm-Gregory, who unexpectedly died in July 2013, much too early in her promising career.

Distribution - So far only known from the type locality.

Diagnosis - Punctate and small, subcircular to pentagonal shell with complete oval foramen perforating the ventral valve. Dorsal valve with long, slightly medially incurved, thick crura with blunt crural processes, no loop. Heavy spiculation within the lophophore and tentacles, and fragile median spiculation in the dorsal mantle.

**Figure 3:**
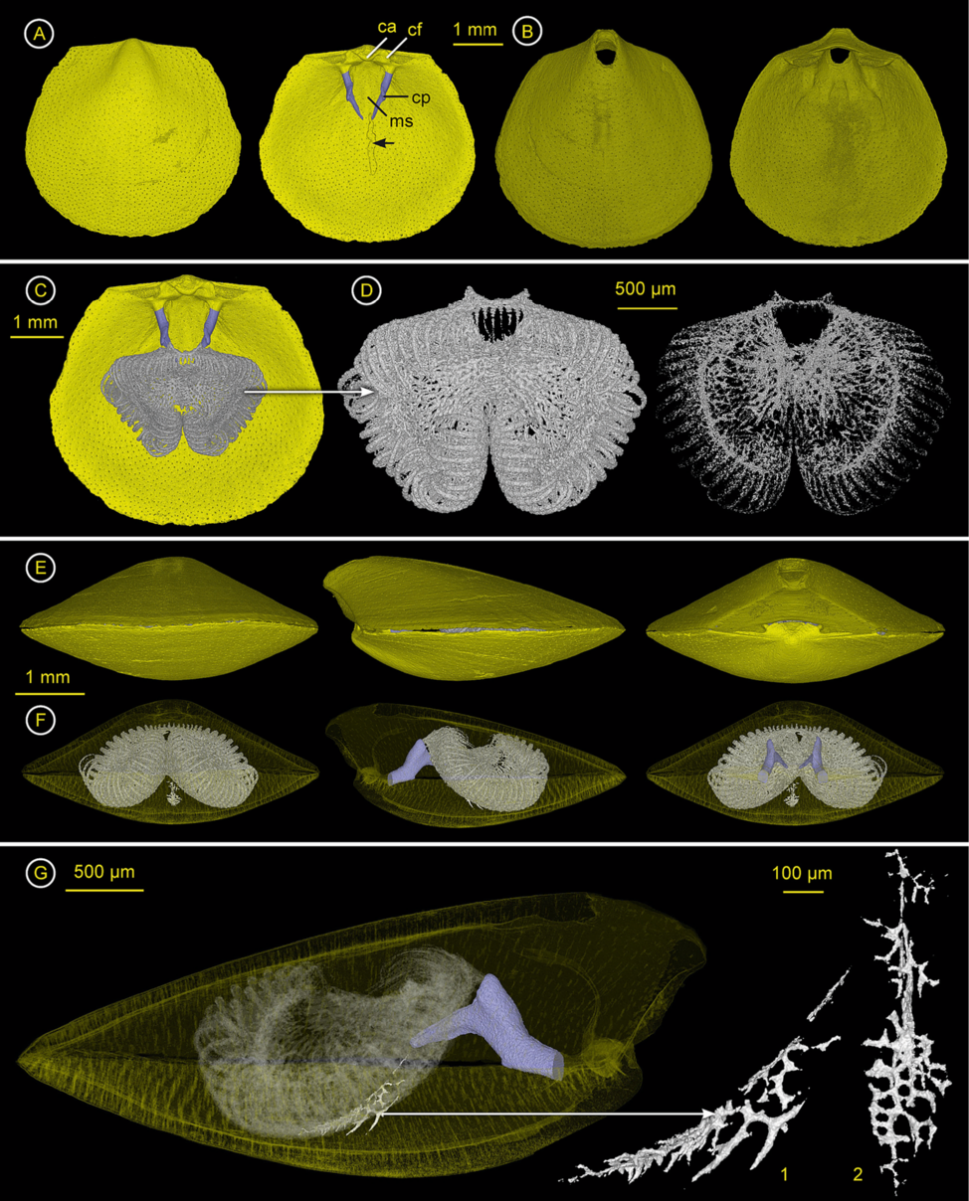
***Rectocalathis schemmgregoryi*** **n. gen., n. sp. (Chlidonophoridae) – ZMB Bra 2254.** Ventral valve = top & dorsal valve = bottom. Click **here** to download a 3D model of *R. schemmgregoryi* n. gen., n. sp. (interactive PDF). **A) Left**: Outside of the dorsal valve. **Right**: Inside of dorsal valve without the lophophoral endoskeleton. Shell with faint depression posteriorly of the center where the dorsal mantle spiculation is located. Crura purple. **B) Left**: Outside of the ventral valve. **Right**: Inside of ventral valve. **C)** Inside of the dorsal valve with lophophoral endoskeleton and spicules of the tentacles. **D) Left**: Ventral view of lophophoral endoskeleton, showing spiculated tentacles and heavy spiculation inside the loops. Posteriorly of the mouth, two symmetrical extensions project towards the crural processes. **Right**: Ventral view of “strongly purged” lophophoral endoskeleton to visualize the geometrical nature. Strongest speculation in the centre of the lophophore, spreading out from anterior of the mouth, to support the lophophoral arms, their strongest spiculation at the base of the tentacles. **E) Left**: Anterior view of whole specimen. **Middle**: Lateral view of whole specimen. **Right**: Posterior view of whole specimen. **F) Left**: Anterior view through transparent shell. **Middle**: Lateral view through transparent shell, **Right**: Posterior view through transparent shell. **G) Left**: Lateral view through transparent shell. Spicules of the dorsal body wall (white) are visible due to transparency of the lophophoral endoskeleton (arrow). **Right**: Magnification of median spicules of the dorsal body wall. 1) lateral view, 2) anterior view.

Description - From ventral, shell subcircular, well rounded anterior margin, lateral margin posteriorly straight and curved towards the front. Anterior commissure rectimarginate or slightly uniplicate, lateral commissure straight (Figure 3A, B, Additional file 4). Dorsal umbo projects beyond the hinge line (Figure 3A), beak long and erect (Figure 3B). From posterior, ventral valve with wide, well differentiated symphytium, triangular, with growth lines (Figure 3E right). Dorsal valve convex, ventral valve wedge-shaped (Figure 3E middle). Both valves posteroaxially keeled, but dorsal valve less sharp than ventral valve (Figure 3E left). Surface with faint costae (n = 14-16), fine growth lines, punctae (anterior margin ~134/mm^2^, in front of protegulum ~69/mm^2^). Shell size in mm L × W × H (dorsal/ventral valve): 4/4.5 × 4/4 × 0.8/1.

Dorsal valve interior (Figure 3A, C). – Hinge line almost straight and wide (0.57 × valve width), sockets large and both ridges almost parallel to hinge line. Outer socket ridges long, narrow, submarginal, inner socket ridges minute, fused with large cardinal flange. Dorsal umbo projects beyond the hinge line, cardinal process large, anteroaxially labiate, laterally fused with cardinal flange. Crura bases broad, crura thick, straight or slightly convergent to the front, from ventral view axes of crural processes slightly rotated from the axis of the crura and descending braches. Surface with punctae, no tubercles, faint median septum in the posterior half, and faint depression posterior of the centre of the valve where the dorsal mantle spiculation is located.

Inside of ventral valve (Figure 3B). – Deltidial plates conjoint, no conjunction line, symphytium abruptly curved posteriorly. Hinge teeth with elongate base, almost parallel to the hinge line, with outer, lateral base submarginal, inner base elongated towards the foramen, distal ends pointed. Surface with punctae, no tubercles. Symmetrical muscle scars within depressed area of the keel between foramen and a thick median groove (mg) within the middle of the posterior half.

Spiculation - Spicules develop within the lophophore, the tentacles and the dorsal mantle (Figure 3C). The spiculation within the schizolophous lophophore is strong, and takes up almost the entire volume of the mantle cavity (Figure 3F, G). It consists of a smaller, centred, radiating network of thick and multibranched spicules and two outer “rings” which support the lophophore with its two arms bearing the strongly spiculated tentacles (Figure 3D). The radiating structure is positioned in between the lophophoral arms as a symmetrical web of spicules supporting all parts of the arms of the lophophore. On the anterior side, the arms of the lophophore are curled ventrally and separated. Posteriorly, they are fused leaving a large opening for the mouth. The spicules of the tentacles are half-cylinder shaped. The lophophoral endoskeleton is positioned on the anterior third of the ventral side of the crura. Posteriorly, two excrescences of the endoskeleton extend next to the broad crural processes to support the endoskeleton’s orientation (Figure 3D). The dorsal mantle exhibits only a faint but distinct spiculation, which is positioned medially just below the tips of the crura, and slopes anteriorly (Figure 3G). The spicules are slender and delicate, and too fragile to support the loop holding the weight of the lophophoral endoskeleton. In contrast to the spiculation in *Eucalathis* sp., these spicules show a distinct morphology composed of two parts (Figure 3G1 and G2). The anterior part is in one plane with the anterior body wall and the posterior part is oriented dorsoventrally, reaching axially into the visceral cavity. While the spicules of the anterior part are short and form a dense network, the spicules of the posterior part are rather long and less branched. The mantle spiculation is not connected to the floor of the valve or to the lophophoral endoskeleton.

Remarks - The spiculation pattern of *Rectocalathis schemmgregoryi* n. gen, n. sp. easily identifies it as a member of Eucalathinae. Especially the spicules of the dorsal mantle epithelium, which are not connected to any other spicules of the internal skeleton, are a characteristic of this group (see also *Eucalathis* sp.). Within Eucalathinae, four genera *Eucalathis* Fischer & Oehlert, 1890*, Bathynanus* Foster, 1974*, Notozyga* Cooper, 1977*,* and *Nanacalathis* Zezina, 1981, are described [25]. All species within these genera have disjunct deltidial plates resulting in a hypothyrid foramen, except *Nanacalathis* which has conjunct deltidial plates forming a mesothyrid foramen. *Rectocalathis schemmgregoryi* n. gen, n. sp. also has conjunct deltidial plates, but an extreme form of an epithyrid foramen with the pedicle opening lying in a subumbonal position on the ventral side of the animal. Comparable to *Nanacalathis* spp., *Rectocalathis schemmgregoryi* n. gen., n. sp. lacks a brachidial loop, but it clearly differs from *Nanacalathis* through its nearly smooth shell. So far, *Rectocalathis schemmgregoryi* n. gen., n. sp. has only been found in the deep sea at a single station during the MANGAN-Expedition of the German research vessel SONNE (SO 205). Same as *Bathynanus* and *Nanacalathis*, it is expected that *Rectocalathis* n. gen. is also restricted to deep water.

### *Eucalathis* sp. (Chlidonophoridae) – ZMB Bra 2351

**Figure 4:**
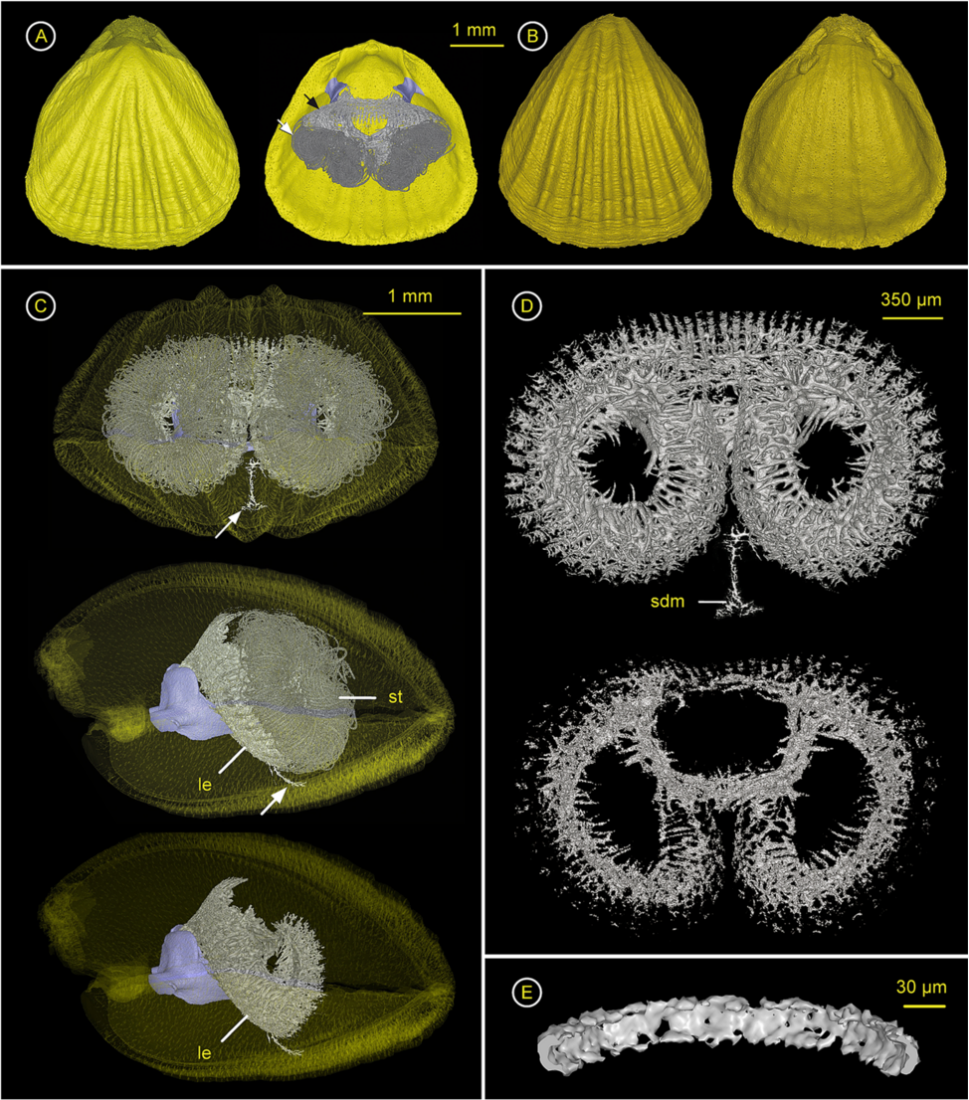
***Eucalathis*** **sp. (Chlidonophoridae) – ZMB Bra 2351.** Ventral valve = top & dorsal valve = bottom. Click **here** to download a 3D model of *Eucalathis* sp. (interactive PDF). **A)** left: Dorsal valve with ventral valve in the background; right: Interior of the dorsal valve, with the short looped brachidium (purple), the endoskeleton of the lophophore (black arrow) with spicules of the tentacles (white arrow). **B)** left: Ventral valve; right: Interior of ventral valve showing the incomplete foramen. **C)** Anterior view (top) and lateral views (middle and bottom) through transparent shell with ventral valve on top, showing the dense lophophoral endoskeleton of the schizolophous lophophore (le), spicules within the tentacles (st) and a few median, slender and rather loose spicules with dorsoventral orientation in the dorsal mantle (arrow). **D)** Anteroventral view of lophophoral endoskeleton with faint spiculation of the dorsal mantle (sdm), bottom: “virtually purged” lophophoral endoskeleton to visualise the geometrical nature, the smaller, inner ring rests on the short looped brachidium. **E)** Cross section through spicules of a tentacle.

Spiculation - Spicules develop within the lophophore, the tentacles (Figure 4A, Additional file 5) and within the dorsal mantle (Figure 4C, D). No spicules were found in the ventral valve (Figure 4B). The lophophoral endoskeleton is massive, dense and large, occupying a considerable volume within the mantle cavity (Figure 4C). It consists of a smaller, inner supportive structure made of large, thick and long, branched spicules and two outer rings which support the lophophore with its two arms bearing the tentacles (Figure 4D). The inner structure with its semicircle-shape fits with a minimal distance on the short looped brachidium and is connected posteriorly to the outer rings of the two arms. Because it fits on the inner side of the brachidium, the heavy lophophoral endoskeleton maintains its position but is also capable of movement due to the gap to the loop. Additionally, posterior outgrowths extend below the broad crural processes to support the endoskeleton’s orientation (Figure 4A). The lophophore bears an alternating row of tentacles. Whereas, the outer tentacles show a rich spiculation throughout (Figure 4C middle), the inner tentacles lack spicules. The conjoint spicules of the outer tentacles are approximately ~30 μm in width and half-cylinder shaped (Figure 4E). The dorsal mantle exhibits only a faint but distinct spiculation, which is positioned medially just below the brachidium at the apical loop, sloping anteriorly (Figure 4C, D). The spicules are slender and delicate, and too fragile to support the loop, which holds the weight of the lophophoral endoskeleton. The mantle spiculation is not connected to the floor of the valve or to the lophophoral endoskeleton.

### *Gryphus vitreus* (Terebratulidae) – ZMB Bra 2261

**Figure 5:**
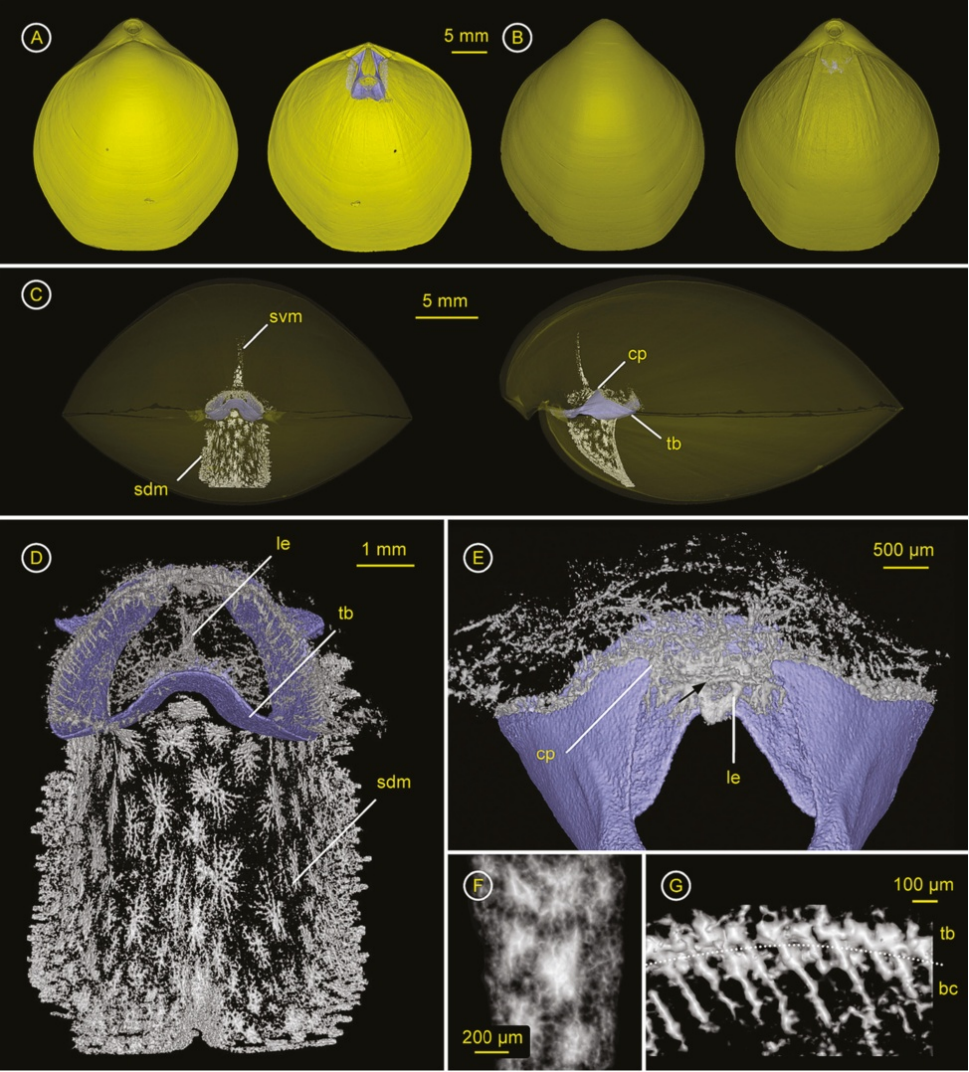
***Gryphus vitreus*** **(Terebratulidae) – ZMB Bra 2261.** Ventral valve = top & dorsal valve = bottom. Click **here** to download a 3D model of *G. vitreus* (interactive PDF). **A)** left: Dorsal valve with ventral valve in the background; right: Interior of the dorsal valve, with the short looped brachidium (purple), the endoskeleton of the lophophore and spiculation in the dorsal mantle. **B)** left: Ventral valve; right: Inside of ventral valve with faint spiculation in the ventral mantle. **C)** Anterior view (left) and lateral view (right) through transparent shell with ventral valve on top, showing the short looped brachidium (purple) with crural processess (cp) and transverse band (tb), dorsal (sdm) and ventral mantle spiculation (svm). **D)** Anteroventral view of lophophoral endoskeleton (le) supported by the brachidium with a ventrally arched transverse band (tb), and the axial posteriorly keeled dorsal mantle spiculation (sdm) with star-shaped spicules (max. ~1.3 mm in diameter). **E)** Posterior view of the dorsal loop (purple) with triangular, and anteriorly converging crural processes (cp) and the lophophoral endoskeleton (le) with a plate-shaped structure and transverse projection (arrow). **F)** Magnification of faint spiculation within the ventral mantle, showing few axial spicules in a loose network. **G)** Magnification of spiculation of the lateral lophophoral endoskeleton with elongated spicules, basal crystals (bc) and tentacle bases (tb).

Spiculation - Spicules develop within the lophophore, and the dorsal and ventral mantle (Figure 5A, B, Additional file 6). Spicules at the tentacle bases and within the tentacles are absent in this specimen. The spiculation within the lophophore is generally faint (Figure 5D), and restricted more or less to the area below the bases of the tentacles, with elongated spicules called basal crystals (Figure 5G). In front of the crural processes the lophophoral spiculation exhibits a compact, plate-shaped structure with a transverse projection (Figure 5E). The spiculation within the ventral valve is faint too, and only few axial spicules were found (Figure 5C, F). In contrast, the spiculation within the dorsal mantle is strong below the short loop of the brachidium (Figure 5C), with plate-shaped, star-shaped and perforated spicules (Figure 5D). No spiculation within the dorsal mantle covering the periphery of the visceral cavity and the pallial vessels towards the margin of the valve could be found.

### *Liothyrella neozelanica* (Terebratulidae) – ZMB Bra 2255

**Figure 6:**
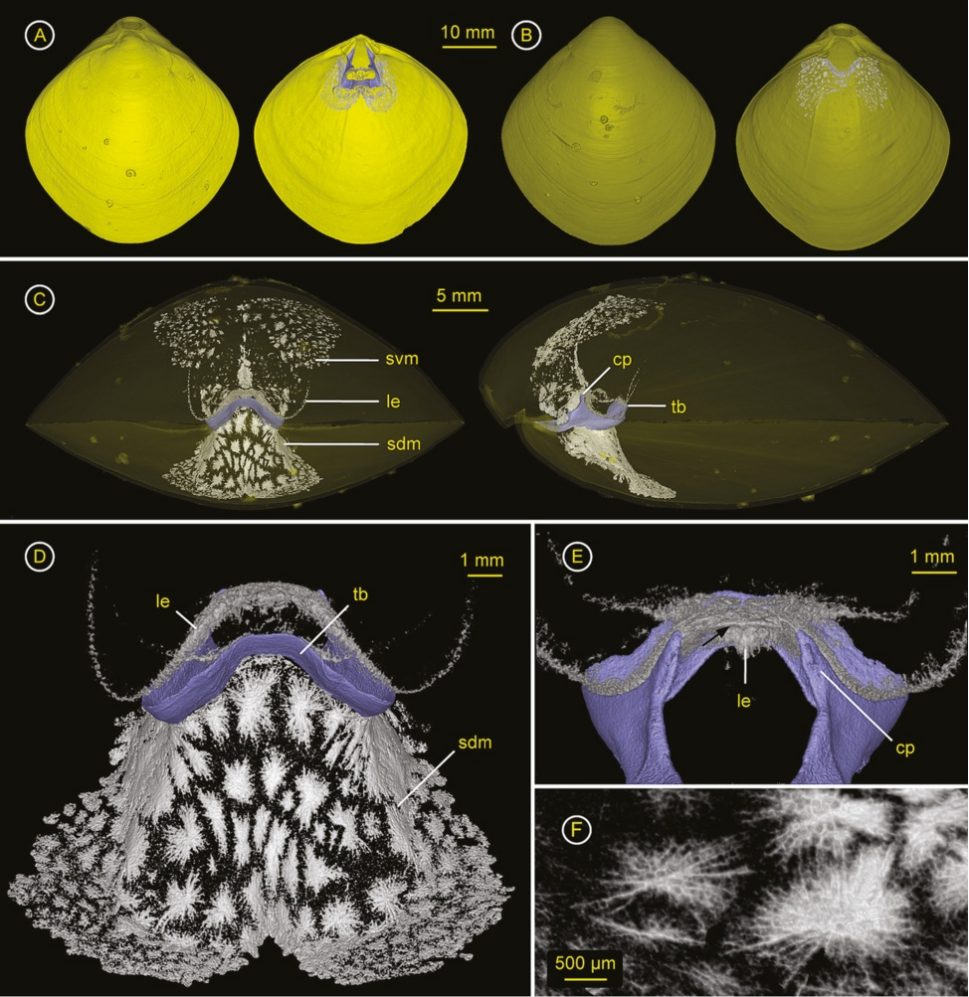
***Liothyrella neozelanica*** **(Terebratulidae) – ZMB Bra 2255.** Ventral valve = top & dorsal valve = bottom. Click **here** to download a 3D model of *L. neozelanica* (interactive PDF). **A)** left: Dorsal valve with Ventral valve in the background; right: Interior of the dorsal valve, with the short looped brachidium (purple), the endoskeleton of the lophophore and spiculation in the dorsal mantle. **B)** left: Ventral valve; right: Inside of ventral valve with spiculation in the ventral mantle. **C)** Anterior view (left) and lateral view (right) through transparent shell with ventral valve on top, showing the short looped brachidium (purple) with crural processess (cp) and transverse band (tb), dorsal (sdm) and ventral mantle spiculation (svm). **D)** Anteroventral view of lophophoral endoskeleton (le) supported by the brachidium with a ventrally arched transverse band (tb), and the axial posteriorly keeled dorsal mantle spiculation (sdm) with star-shaped spicules (max. ~1.3 mm in diameter). **E)** Posterior view on the brachial loop (purple) with triangular, and anteriorly converging crural processes (cp) and the lophophoral endoskeleton (le) with a plate-shaped structure and transverse projection (arrow). **F)** Magnification of spicules from the anterior part of the dorsal body wall, showing the starlike nature, sizes range from ~0.5 mm to ~2.5 mm in diameter and max. ~150 μm in thickness.

Spiculation - *L. neozelanica* develops spicules within the lophophore, ventral and dorsal body wall (Figure 6A, B, Additional file 7). The mineralisation within the plectolophous lophophore is minimal (Figure 6D, E). Only in the posteroaxial part of the lophophoral endoskeleton more spicules are developed which cluster. Here, as in *G. vitreus*, a compact, plate-shaped structure with a transverse projection can be found (Figure 6E). The shape of single spicules within the arms of the lophophore could not be identified unambiguously. The spiculation within the dorsal and ventral valve is unequal (Figure 6C). While the ventral valve exhibits a very faint and loose network of more slender, branched, and irregularly shaped spicules, the body wall of the dorsal valve shows a rich spiculation (Figure 6D). Large and thick, star-shaped and closely spaced spicules cover the visceral cavity below the brachidium (Figure 6F). Especially laterally, close to the brachidium, the spiculation is significantly stronger than anywhere else within the mantle. Spiculation within the dorsal mantle covering the periphery of the visceral cavity and the pallial vessels towards the margin of the valve, could not be found.

### Full data sets and additional material

Figures and interactive 3D models of additional 5 species with spicules (*Laqueus rubellus* (Sowerby, 1846) *–* Additional files 8 and 9, *M. truncata –* Additional files 10 and 11, *P. anomioides –* Additional files 12 and 13, *Pumilus antiquatus* Atkins, 1958 *–* Additional files 14 and 15, *P. atlantica –* Additional files 16 and 17) can be accessed as Online Supplemental Material, as well as a phylogenetic tree and tables concerning the scan parameters and morphometric analyses of all species of the initial study.

## Discussion

### Applicability of micro-computed tomography for the study of modern brachiopods

Scanning modern brachiopods with an X-ray micro-computed tomograph (μCT) creates naturally aligned virtual sections (2D) and interactive 3D models. μCT is a unique tool for obtaining and visualising structural and positional data of the spiculation and the inner shell morphology of modern brachiopods. The present μCT data visualises the endoskeleton and the shell with high accuracy and natural orientation. In a short period of time, the endoskeletal spiculation in 10 out of 19 investigated genera was detected by analyzing stacks of 2 dimensional images. The scanning and reconstruction is a clean and quick procedure, whereas the subsequent virtual sectioning and colouration necessary to create 3D models is much more time consuming, but is particularly important for understanding the three dimensional architecture of structures, linking colours to regions of interest and it results in an unlimited set of views. Hard parts of the animals can be easily defined and marked, whereas soft tissue visualization needs staining.

With the achieved resolution of 10 microns the anatomy of single spicules and differences in the surface texture (e.g. muscle scars) can be visualised. However, the quality of images depends significantly on the rendering algorithm that is applied, which is in turn linked to the computing power and total operation time. In this study the volume renderer PHONG and an isosurface renderer were used. The latter does not affect the CPU as much as the volume render and yields good results for simulating transparency. The chosen scan parameters represent appropriate balances between so called “fast scans” (~15 minutes) and scans of longer duration (>50 minutes) which resulted in data of more than 20 GB / file). The presence of minute structures such as spicules and punctae canals could easily be detected with the scan parameters that were applied, in whole mount scans of any specimen size.

μCT (Phoenix Nanotom XS 180 NF) produces high resolution images of brachiopods visualising their mineralized exo- and endoskeleton and allows rotational analysis of the specimen. Depending on the condition of the animal and the scan duration (scan parameters, such as number of images, timing, “average and skip”) μCT images are comparable to SEM images by showing the full set of informative characters of the shell, e.g. muscle scars, punctae, growth lines, costae, tubercles, septae, spicules, teeth and sockets, crura and brachidium. However, in regard to the resolution it is less effective than scanning electron microscopy (SEM), hence SEM would be the first method of choice for surface-sensitive investigations, especially when resolutions of only a few microns or less are needed. None of the specimens investigated in this study were physically damaged through μCT scanning. As a result, after being scanned the specimen of *Eucalathis* sp. was successfully included in a DNA sequencing analysis which underlines the compatibility of μCT and molecular analyses. However, there is no certainty about a maximum radiation dose for successful post-scan DNA sequencing and therefore, rather short scans and immediate DNA amplification are recommended.

### What is the function of a brachiopod’s endoskeleton?

Speculation about the function of spiculation has been fuelled by a general debate concerning the vulnerability of brachiopods [26]. Their generally poor soft tissue yield results in little predation pressure, especially when the tissue contains spicules or toxins [27,28]. However, it is known that modern echinoderms, gastropods and crabs certainly penetrate the shell and do feed on brachiopods [29,30 and references therein]. The strength of the brachiopod shell is more affected by the thickness and ribbing, rather than by the biconvexity or presence of punctae [31]. However, organic matter is inaccessible to predators when placed within the punctae and similarly the internal tissue is not palatable when containing high amounts of inorganic spicules. Analyses of tissue of modern brachiopods and their value to predators revealed a higher inorganic content in the tissue of punctate than the impunctate brachiopods [32], which is in accordance with the observations in this study. Therefore, the co-occurence of spiculation and punctation may be referred to as non-chemical responses to predatory pressure.

Another criterion is the mechanical support for the soft tissue, especially of the lophophore. Mechanical tests with *Terebratulina unguicula* (Carpenter, 1864) revealed that a mean elastic modulus for spiculated tissues is more than three times greater than for artificially decalcified tissues [33]. In this study, all of the “short looped” specimens with little exoskeletal support of the lophophore (brachidium) exhibit spicules within the lophophore. Certain load-bearing features of the endoskeleton can be identified. The design of these structures suggests a perfect cooperation with the brachidium to maintain function and orientation of the lophophore with its protruding arms, as observed in the posterior excrescences of the endoskeleton in *Eucalathis* sp. and *Rectocalathis schemmgregoryi* n. gen., n. sp. or in the distinct thick and plate-shaped structures for load-bearing in *G. vitreus* and *L. neozelanica*. The conspicuous load-bearing structure of *G. vitreus* and *L. neozelanica* can be addressed as an additional synapomorphic character of the family Terebratulidae (see Additional file 18: Figure S6. Phylogeny), identified due to this nondestructive μCT approach. A massive, area-wide spiculation within soft parts offers either mechanical support or fulfils a potent anti-predator function, or both. It is questionable whether the weak spiculation in the dorsal body wall of *Eucalathis* sp. and *Rectocalathis schemmgregoryi* n. gen., n. sp. serves as mechanical support. The anatomy suggests a regulation of the coelomic cavity pressure in interaction with muscles. Nonetheless, the weak spiculation in the dorsal body wall as well as in the specialized lophophoral endoskeleton indicates the close phylogenetic relationship of both species (see Additional file 16: Figure S6. Phylogeny)

## Conclusion

The aim of this μCT study was to depict the profound differences of the hard parts and shell anatomy of modern brachiopods and to establish μCT scan and subsequent data processing protocols suitable for specimens preserved in alcohol. Micro-CT is an excellent non-destructive tool for visualizing the calcified exo- and endoskeleton of brachiopods. Acquired data on spiculation delivers a substantial contribution to formerly available documentations of brachiopod hard parts (endoskeleton) due to its visualization in its entirety. The phylogenetic relevance of spiculation has been indicated, with promising new informative characters. However extensive sampling is necessary to verify these findings and to discover intra-specific variability of spiculation.

## Materials and methods

Representatives of 17 brachiopod genera covering all modern articulated subgroups and two representatives of inarticulated craniids were scanned for morphological analysis. The results from specimens with spicules are shown in this paper or can be accessed in the Supplemental Material. All specimens were selected from either the historical wet collection of the Museum für Naturkunde Berlin, Germany or from recently collected samples. For specimens used in this study see Table 1 and Additional file 1: Table S1. All specimens were stored in 75 - 80% ethanol (EtOH). For scanning, the specimen’s valves were slightly opened with a needle and transferred gradually to distilled water. The scanning was performed dry to prevent soft tissue from moving, especially of the lophophore and the tentacles. For scanning the calcareous shell, it made no difference whether the prefixation was performed in alcohol, formaldehyde or Bouin’s solution.

The specimens were X-rayed using a Phoenix Nanotom XS 180 NF (max. operating data 180kv, 1,0 mA, 15W – GE Sensing & Inspection Technologies GmbH, Wunstorf, Germany). The samples were placed in custom built carriers made of centrifuge tubes of 1,5 ml, 25 ml and 50 ml or Kautex bottles with a foam plastic inlay to prevent movement artefacts. Once the whole geometry of the specimen was scanned the X-ray images were reconstructed with “datos|x – reconstruction 1.5.0.22” (GE Inspection Technologies) using 4 cluster PCs to create the actual working file. Minimal movement of the specimen within the carrier (along distinct axes – not rotation) could be subtracted prior to reconstruction by aligning the first and an additionally taken image (number of images +1) after a full 360° rotation (tool name: scan optimisation with first and last image). For scanning parameters used in this study see Table 3 and Additional file 19: Table S3.

**Table 3:** **Scan parameters used in this study**

**Species**	**Images**	**Voltage (kV)**	**Current (μA)**	**Timing value (ms)**	**Averaging & Skip**	**Magnification**	**Scan Time ~ (min)**
*T. retusa*	900	90	200	750	2 – 1	5,7	45
*R. schemmgregoryi* n. gen., n. sp.	900	60	100	750	2 – 1	17,6	45
*Eucalathis* sp.	900	60	130	750	2 – 1	13,2	45
*G. vitreus*	900	80	280	750	2 – 1	3,1	45
*L. neozelanica*	900	80	280	750	2 – 1	2,4	45

For scan parameters of the remaining species of the initial study (see Additional file 19: Table S3).

Image processing of the initial 3D reconstruction with earmarking of the “regions of interests”, cleaning of the volume, specimen analyses and morphometries was carried out on a Dell Workstation (Dell Precision T7500, Intel Xeon E5530, 2.4 GHz; graphics card: NVIDIA GeForce GTX 285; operating system: Microsoft Windows XP Professional x64 Edition) using the software “Volume Graphics Studio Max 2.1” (Volume Graphics GmbH, Heidelberg, Germany, www.volumegraphics.com). The reconstructions were virtually sectioned to illustrate key features. Images (also 360° full HD (1920×1080) movie clips, data not shown, for details please contact the authors) were obtained using an isosurface or volume (PHONG) rendering algorithm. To illustrate the natural position of the spiculation and internal shell features the isosurface render algorithm was used to simulate transparency of the shell.

For subsequent data processing mostly open source software was used. As for the 3D interactive models, the open source software “MeshLab 1.3.2” (MeshLab, Visual Computing Lab – ISTI – CNR; http://meshlab.sourceforge.net/) for cleaning and down sampling the meshes and “DAZ Studio 4.5.0.114 Standart”(DAZ Productions, Inc., dba DAZ 3D; 224 South 200 West #250, Salt Lake City, UT 84101 USA; http://www.daz3d.com/) for creating the u3d file format were used. The final 3D interactive pdf file was created using the commercial software Adobe Acrobat 8 Professional 8.1.0. (Adobe® Systems Incorporated, San José, CA).

## Additional files

Additional file 1: Table S1. Species used in the initial study. Species of a subgroup are listed in alphabetical order. Additional file 2: Table S2. Volume analyses and morphometric measurements of all species of the initial study using VG Studio Max 2.1. Abbr.: DV = dorsal valve, VV = ventral valve, Spic Loph = spiculation within lophophore, S*pic* DV/VV spiculation within mantle of dorsal/ventral valve, Vol = Volume, Vol. total = Vol. VV + DV + Loph + Spic, × = no spiculation, na = not applicable. Additional file 3: 3D model.
*Terebratulina retusa*. Additional file 4: 3D model.
*Rectocalathis schemmgregoryi* n.gen., n. sp. Additional file 5: 3D model.
*Eucalathis* sp. Additional file 6: 3D model.
*Gryphus vitreus*. Additional file 7: 3D model.
*Liothyrella neozelanica.*
Additional file 8: Figure S1.
*Laqueus rubellus*. Additional file 9: 3D model.
*Laqueus rubellus*. Additional file 10: Figure S2.
*Megerlia truncata*. Additional file 11: 3D model.
*Megerlia truncata*. Additional file 12: Figure S3.
*Platidia anomioides*. Additional file 13: 3D model.
*Platidia animioides*. Additional file 14: Figure S4.
*Pumilus antiquatus*. Additional file 15: 3D model.
*Pumilus aquaticus.*
Additional file 16: Figure S5.
*Pajaudina atlantica*. Additional file 17: 3D model.
*Pajaudina atlantica*. Additional file 18: Figure S6. Phylogeny. Additional file 19: Table S3. Scanning parameters of all species of the initial study. Specimens are listed in alphabetical order. *P. atlantica* 3 was scanned twice using different parameters.
