# Supplementary material for: Overcoming the fragility – X-ray computed micro-tomography elucidates brachiopod endoskeletons
Source: Front Zool. 2014 Sep 27;11:65. doi: 10.1186/s12983-014-0065-x (PMC4312452; doi:10.1186/s12983-014-0065-x)
Supplement: Additional file 1: Table S1. — Species used in the initial study. Species of a subgroup are listed in alphabetical order. [file 12983_2014_65_MOESM1_ESM.pdf]

Order	Species	Location	Date	GPS	Depth[m]	Collector	ZMB
Terebratulida („long loops“)							
	Calloria inconspicua (Sowerby, 1846)	Pudding Island, Otago Peninsula, NZ	02.02.2003	044°00'00'' S, 168°00'00'' E	0.5	C. Lüter	Bra 2246
	Dallina septigera (Lovén, 1846)	DTE, NW of Scotland, St. 3 (also St. 4)	05.08.1898	057°26'00'' N, 001°28'00'' W	79	DTE	Bra 2260
	Laqueus rubellus (Sowerby, 1846)	Hakodate, Japan	–	041°70'00'' N, 140°75'00'' E	–	F. M. Hilgendorf	Bra 2259
	Megathiris detruncata (Gmelin, 1789)	Canico, Madeira, cave, Mediterranean	10.11.1959	032°40'00'' N, 016.50'00'' W	12	P. Wirtz	Bra 2257
	Megerlia truncata (Linnaeus, 1767)	La Calle, Mediterranean	–	–	–	A. O. Kowalewski	Bra 2258
	Platidia sp. (gen. Costa, 1852)	SO208 / St. 17, W-Pacific, W of Costa Rica	21.07.2010	008°44.69' N, 090°43.54 W	3300	N. Furchheim	Bra 2256
	Pumilus antiquatus Atkins, 1958	Pudding Island, Otago Peninsula, NZ	02.02.2003	044°00'00'' S, 168°00'00'' E	0.5	C. Lüter	Bra 2252
	Terebratella sanguinea (Leach, 1814)	Doubtful Sound, South Island, NZ	12.06.1996	045°22'03'' S, 167°00'70'' E	–	P. Meredith	Bra 2247
Terebratulida („short loops“)							
	Eucalathis sp. (gen. Fischer & Œhlert, 1890)	RV MSM19 / 1076-1, S-Atlantic, SW of S. Africa	04.12.2011	040°22.46' S, 014°53.77' E	2018	N. Furchheim	SEM
	Gryphus vitreus (Born, 1778)	Neapel, Mediterranean	–	–	–	F. Blochmann	Bra 2261
	Liothyrella neozelanica (Thomson, 1918)	Doubtful Sound, Bauza Island, NZ	12.06.1996	045°22'03'' S, 167°00'70'' E	–	P. Meredith	Bra 2255
	Terebratulina retusa (Linnaeus, 1958)	Norway	–	–	–	Sars	Bra 2253
	Rectocalathis schemmgregoryi n. gen., n. sp.	SO205 / St. 40, NE Pacific	05.05.2010	011°47'56''N, 116°49'85''W	3954	–	Bra 2254
Rhynchonellida							
	Hemithiris psittacea (Gmelin, 1790)	Balsfjord Berg, Tromsö, Norway	July, 1998	069°34'07'' N, 018°54'03'' E	64	C. Wolter	Bra 2245
	Notosaria nigricans (Sowerby, 1846)	Karitane Point, NZ	02.05.1996	043°34'00'' S, 172°37'50'' E	10	S. Carson, J. Fyfe	Bra 2244
Thecideida							
	Pajaudina atlantica 1 Logan, 1988	Puerto Naos "Arrecife de la casa", NE Atlantic	24.06.2006	028°35'20'' N, 017°54'62'' W	20	G. Maghon, J. Hoffmann	Bra 2248
	Pajaudina atlantica 2 Logan, 1988	Puerto Naos "Arrecife de la casa", NE Atlantic	24.06.2006	028°35'20'' N, 017°54'62'' W	20	G. Maghon, J. Hoffmann	Bra 2249
	Pajaudina atlantica 3 Logan, 1988	Puerto Naos "Arrecife de la casa", NE Atlantic	24.06.2006	028°35'20'' N, 017°54'62'' W	20	G. Maghon, J. Hoffmann	Bra 2250
	Thecidellina sp. (gen. Thompon, 1915)	Osprey Reef, North Horn, Coral Sea, AUS	3.12.2009	013°48‘05“ S, 146°32‘46“ E	6	C. Lüter	Bra 2251
Craniida (inarticulate brachiopods)							
	Neoancistrocrania norfolki Laurin, 1992	Norfolk 2, DW 2024,	27.10.2003	023°27'92'' S, 167°50'90'' E	370	–	Bra 2242
	Novocrania anomala (Müller, 1776)	Tyreholmen, Danmark	–	054°53'31'' N, 012°2'45.9'' E	–	F. Blochmann	Bra 2243
