# Supplementary material for: Overcoming the fragility – X-ray computed micro-tomography elucidates brachiopod endoskeletons
Source: Front Zool. 2014 Sep 27;11:65. doi: 10.1186/s12983-014-0065-x (PMC4312452; doi:10.1186/s12983-014-0065-x)
Supplement: Additional file 2: Table S2. — Volume analyses and morphometric measurements of all species of the initial study using VG Studio Max 2.1. Abbr.: DV = dorsal valve, VV = ventral valve, Spic Loph = spiculation within lophophore, Spic DV/VV spiculation within mantle of dorsal/ventral valve, Vol = Volume, Vol. total = Vol. VV + DV + Loph + Spic, × = no spiculation, na = not applicable. [file 12983_2014_65_MOESM2_ESM.pdf]

Specimen	Vol. total [mm³]	Vol. VV [mm³]	Vol. DV [mm³]	Vol. Spic. Loph [mm³]	Vol. spic. DV/ VV [mm³]	Length VV [mm]	Length DV [mm]	Width VV [mm]	Width DV [mm]	Height VV [mm]	Height DV [mm]
Calloria inconspicua (Sowerby, 1846)	565,05	328,5	230,8	x	x / x	20,8	17,2	18,8	18,8	6,1	5,5
Dallina septigera (Lovén, 1846)	653,22	383,9	259,5	x	x / x	26,4	22,2	19,3	19,3	11,4	5,0
Laqueus rubellus (Sowerby, 1846)	310,42	158,0	144,0	0,1	1,59 / 1,37	22,2	19,7	17,9	17,9	6,8	5,4
Megathiris detruncata (Gmelin, 1789)	24,54	14,6	8,4	x	x / x	4,1	4,1	8,1	8,1	2,2	1,2
Megerlia truncata (Linnaeus, 1767)	60,07	26,9	24,9	1,7	2,6 / 3	9,5	8,4	11,7	11,7	2,1	1,4
Platidia anomioides (Scacchi & Philippi, 1844)	4,25	2,5	1,7	0,2	0,02 / x	4,9	4,5	5,4	5,4	1,1	0,3
Pumilus antiquatus Atkins, 1958	6,11	3,6	2,4	0,1	x / x	4,3	3,5	3,4	3,0	1,2	1,6
Terebratella sanguinea (Leach, 1814)	882,75	534,3	337,2	x	x / x	30,6	26,6	31,7	31,6	8,2	5,9
Eucalathis sp. (gen. Fischer & Öhlert, 1890)	na	na	na	na	na	4,3	3,8	4	4	1,5	1,1
Gryphus vitreus (Born, 1778)	682,22	441,2	223,3	5,0	7,1 / 1,72	32,6	29,3	28,5	28,5	10,8	7,6
Liothyrella neozelanica (Thomson, 1918)	1399,05	772,7	551,0	7,6	34 / 26	42,3	38.67	40,0	40,0	13,0	7,6
Terebratulina retusa (Linnaeus, 1958)	194,2	104,5	88,4	1,8	1,1 / 1,23	18,2	16,4	14,7	14,7	5,0	3,6
Rectocalathis schemmgregoryi n. gen., n. sp	3,77	2,0	1,5	0,2	0,01/ x	4,5	4,0	4,4	4,4	1,0	0,8
Hemithiris psittacea (Gmelin, 1790)	317,38	175,6	141,0	x	x / x	15,5	18,6	18,7	18,7	5,3	5,8
Notosaria nigricans (Sowerby, 1846)	331,9	170,0	161,0	x	x / x	17,5	14,9	20,0	20,0	4,3	5,3
Pajaudina atlantica 1 male Logan, 1988	78,7	54,7	24,0	x	x / 0,16	7,2	5,2	5,9	5,4	3,1	1,3
Thecidellina sp. (gen. Thomson, 1915)	30,1	21,3	8,8	x	x / x	6	5	5	4,9	3	1,1
Neoancistrocrania norfolki Laurin, 1992	1072,7	868,0	192,0	x	x / x	15,6	15,6	17,8	16,8	9,4	4,9
Novocrania anomala (Müller, 1776)	25,24	0,0	25,2	x	x / x	x	8,6	x	9,0	x	2,6
