# Supplementary figures and images for: Overcoming the fragility – X-ray computed micro-tomography elucidates brachiopod endoskeletons
Source: Front Zool. 2014 Sep 27;11:65. doi: 10.1186/s12983-014-0065-x (PMC4312452; doi:10.1186/s12983-014-0065-x)

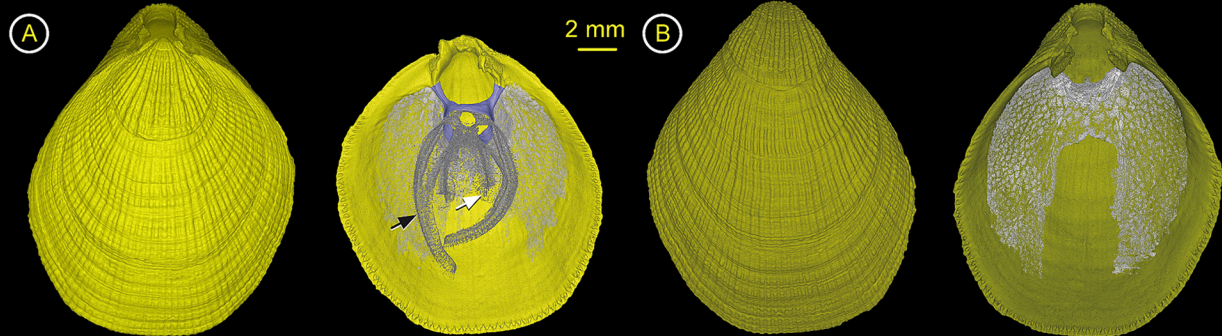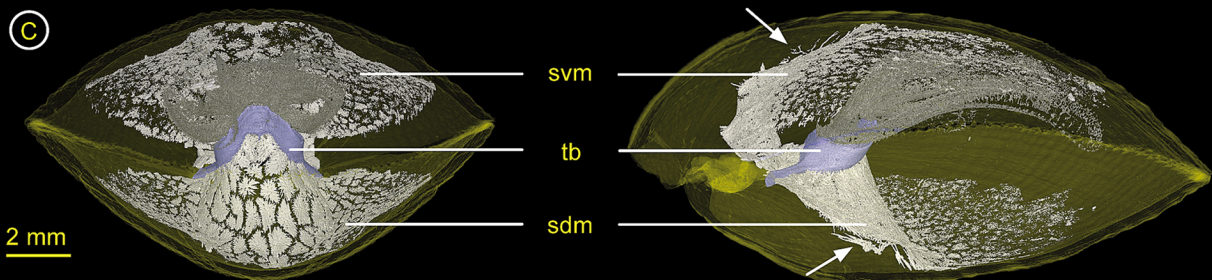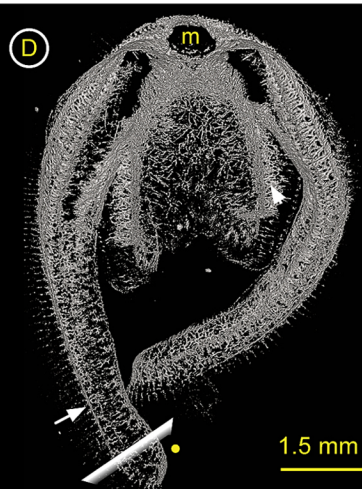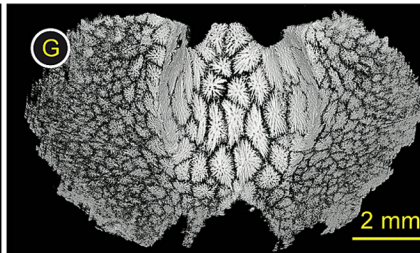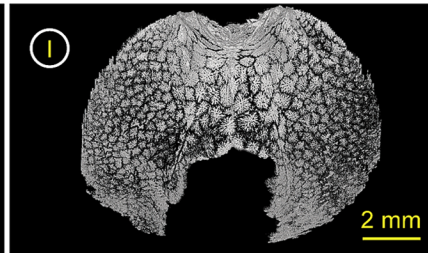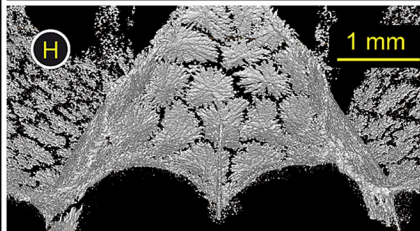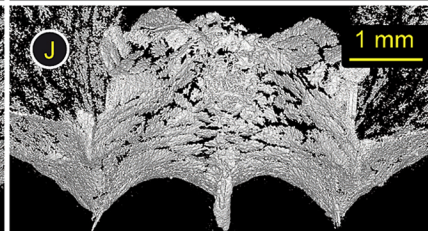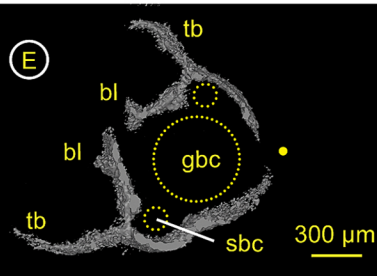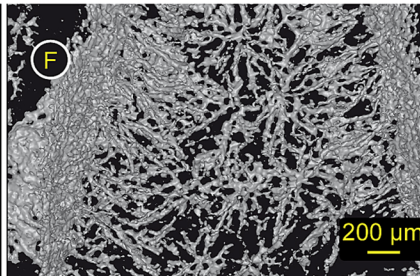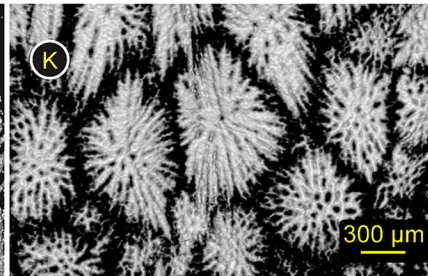

Supplement: Additional file 3: 3D model. — Terebratulina retusa. [file 12983_2014_65_MOESM3_ESM.pdf]

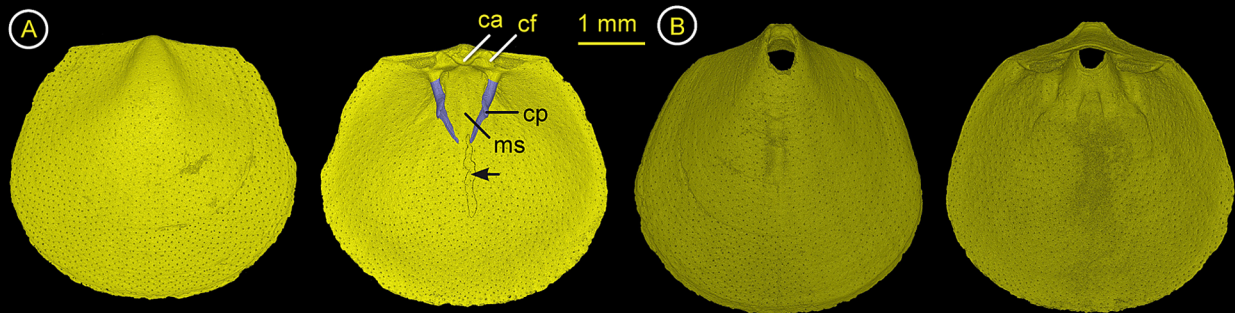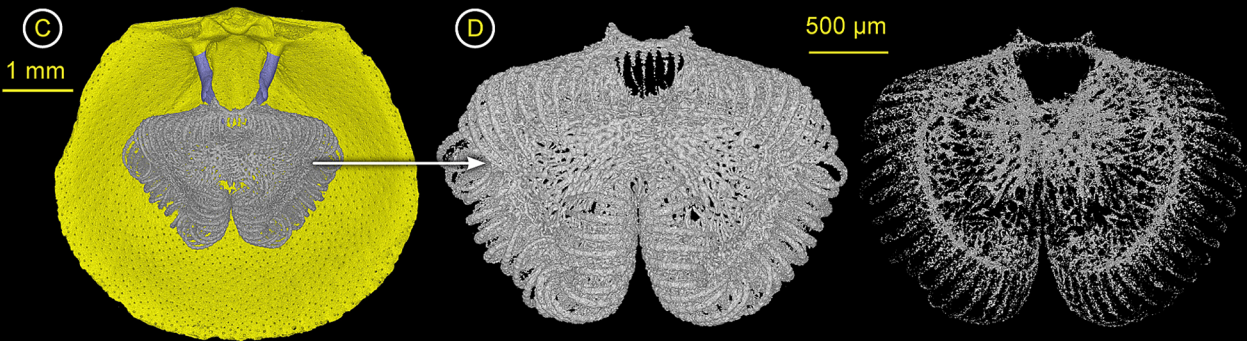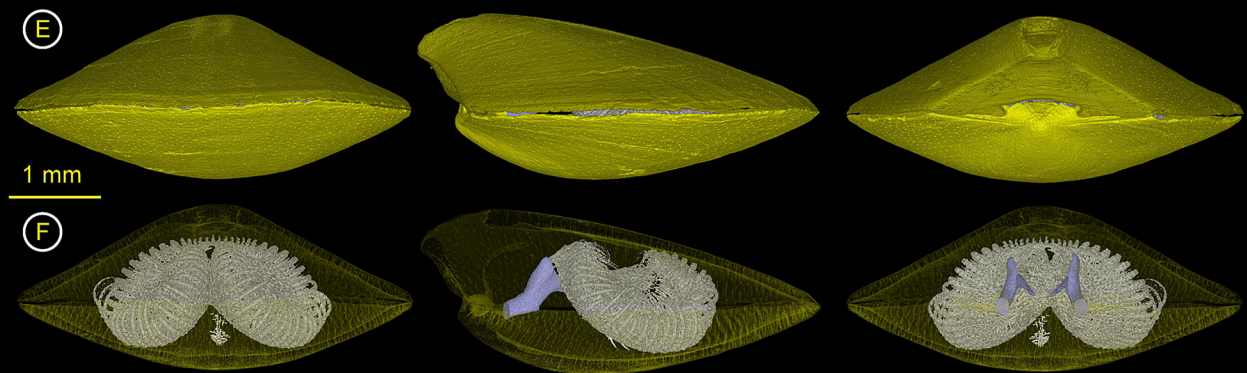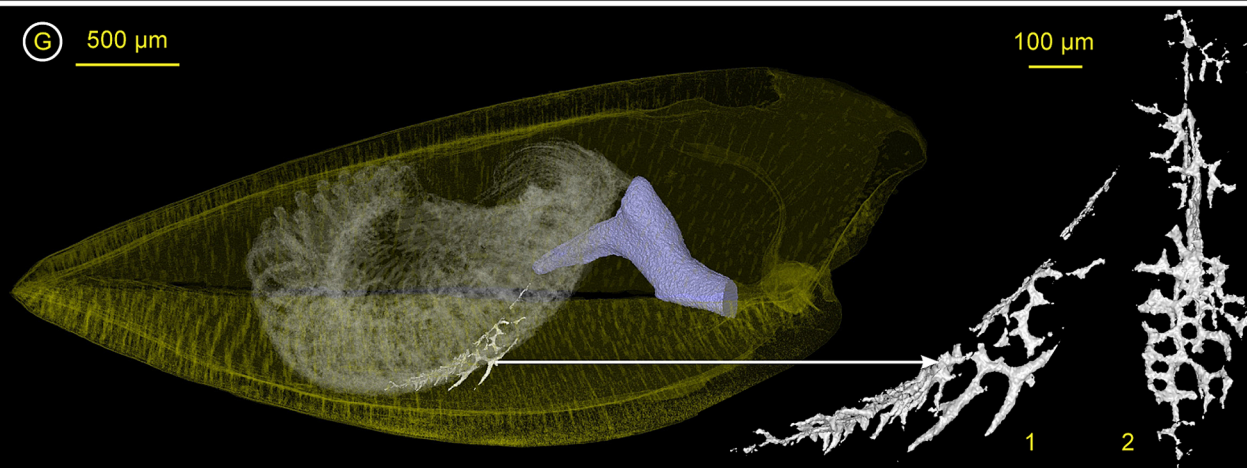

Supplement: Additional file 4: 3D model. — Rectocalathis schemmgregoryi n.gen., n. sp. [file 12983_2014_65_MOESM4_ESM.pdf]

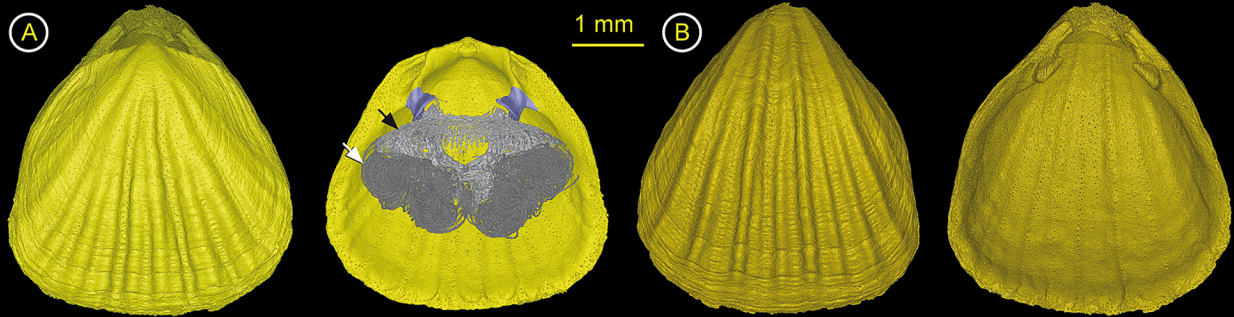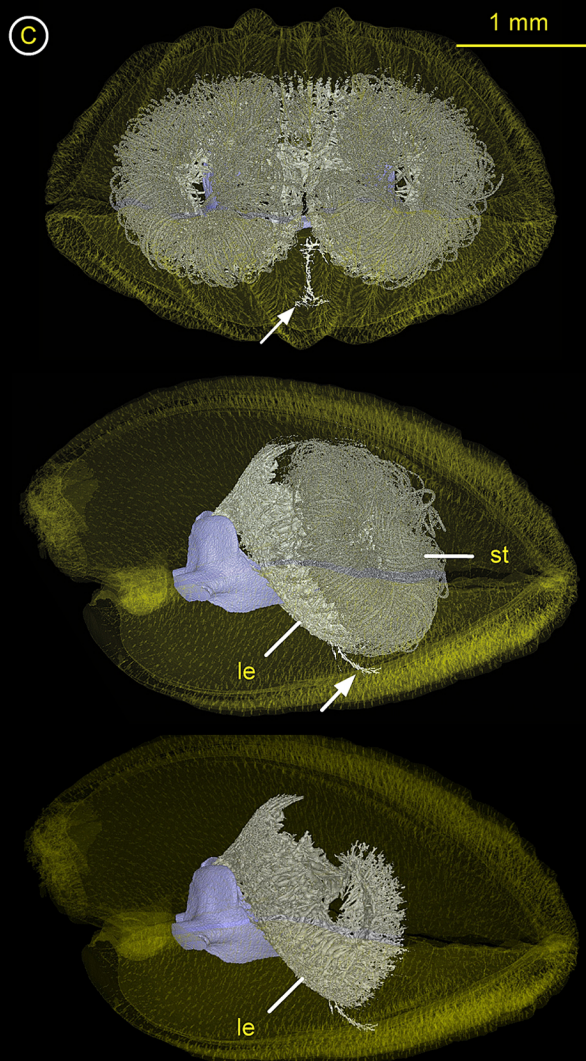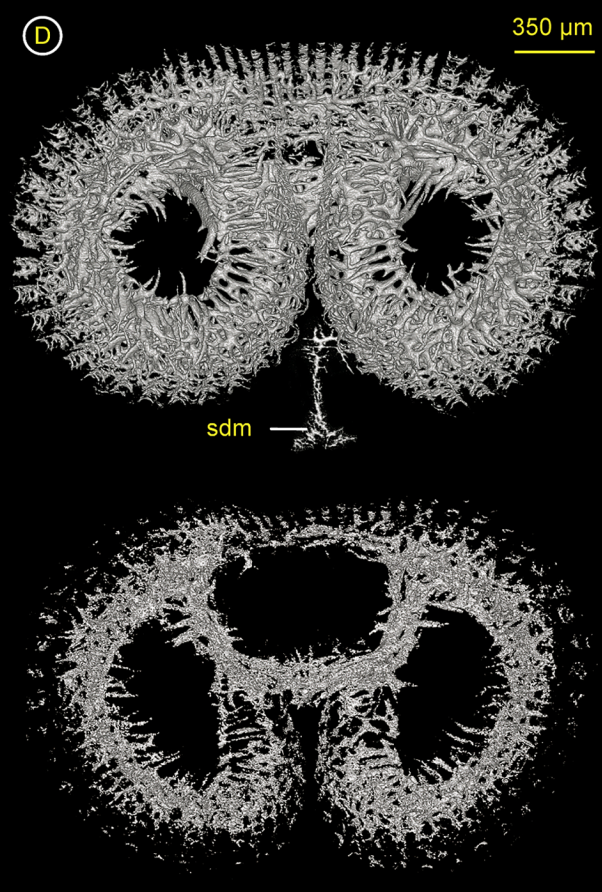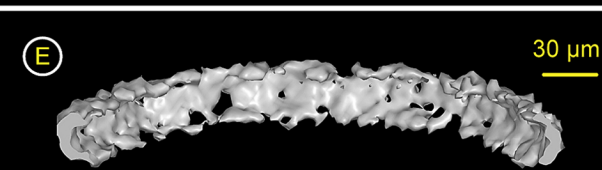

Supplement: Additional file 5: 3D model. — Eucalathis sp. [file 12983_2014_65_MOESM5_ESM.pdf]

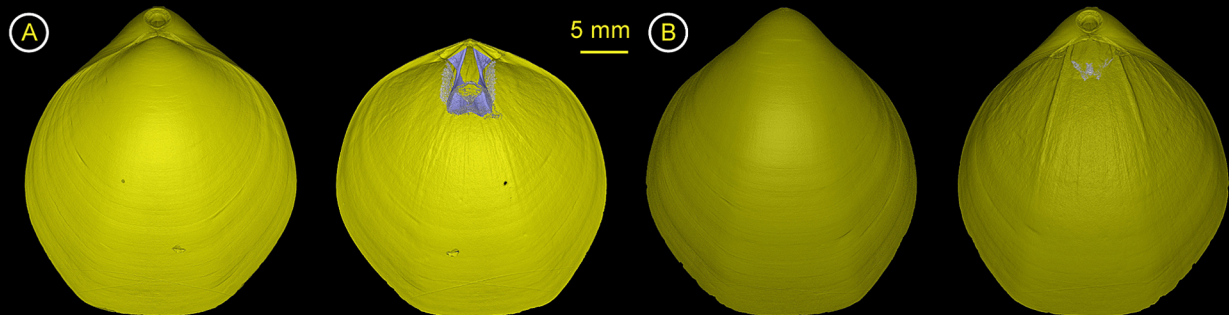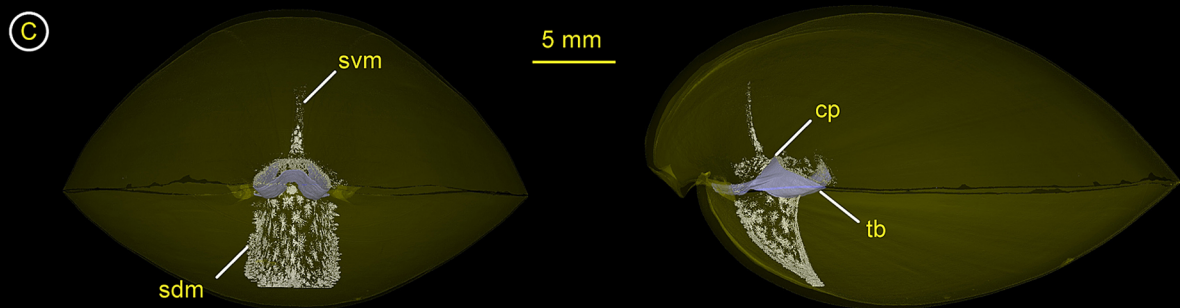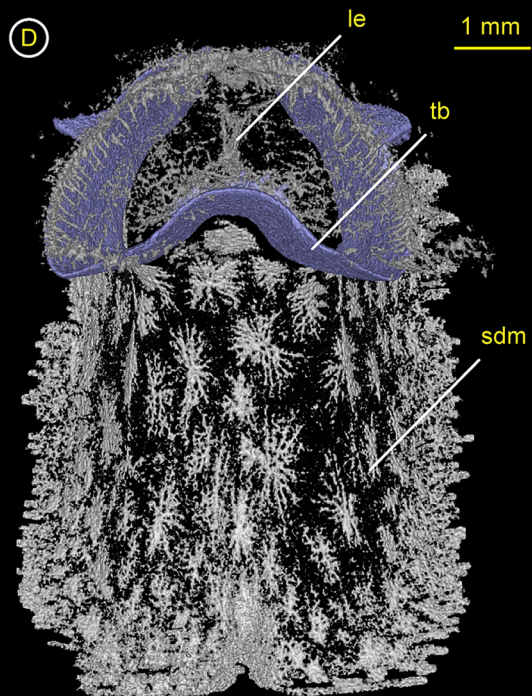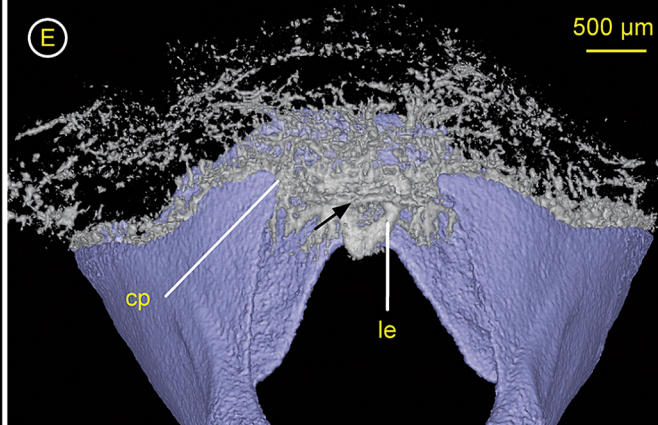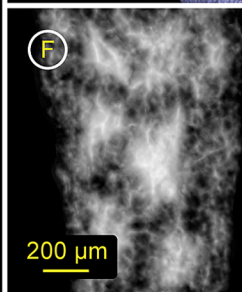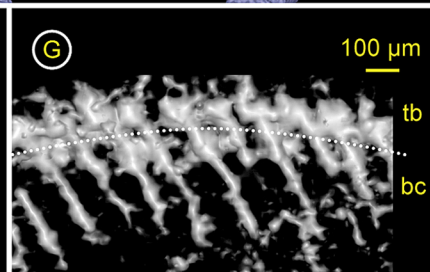

Supplement: Additional file 6: 3D model. — Gryphus vitreus. [file 12983_2014_65_MOESM6_ESM.pdf]

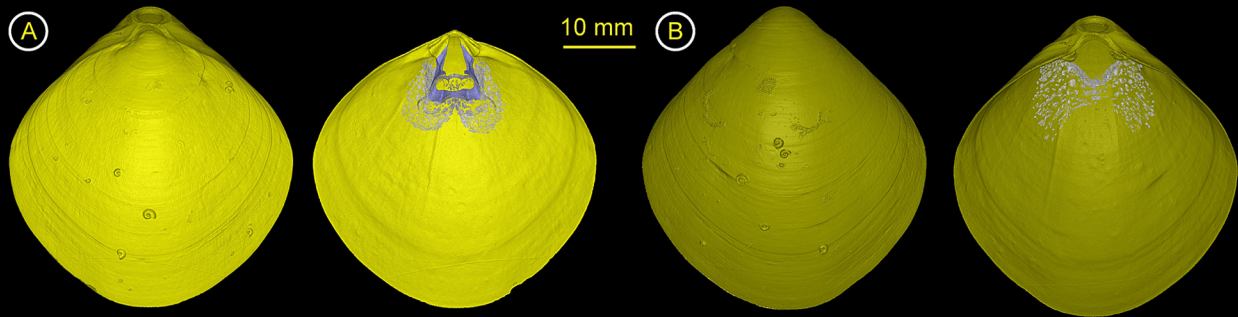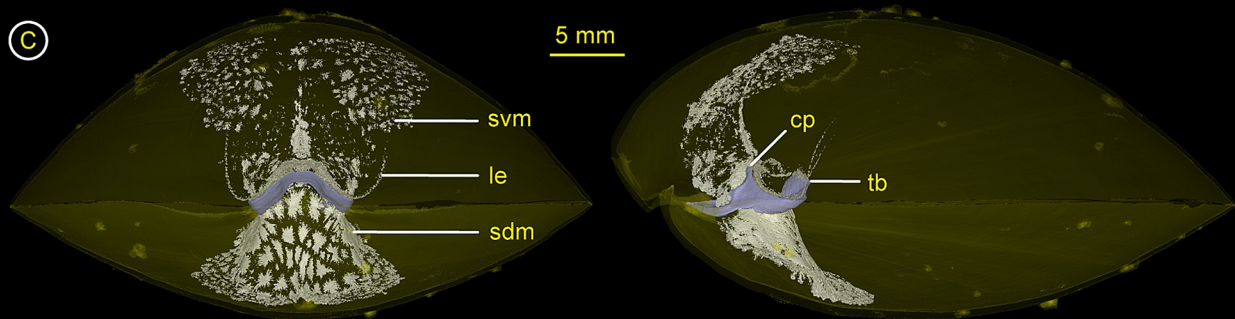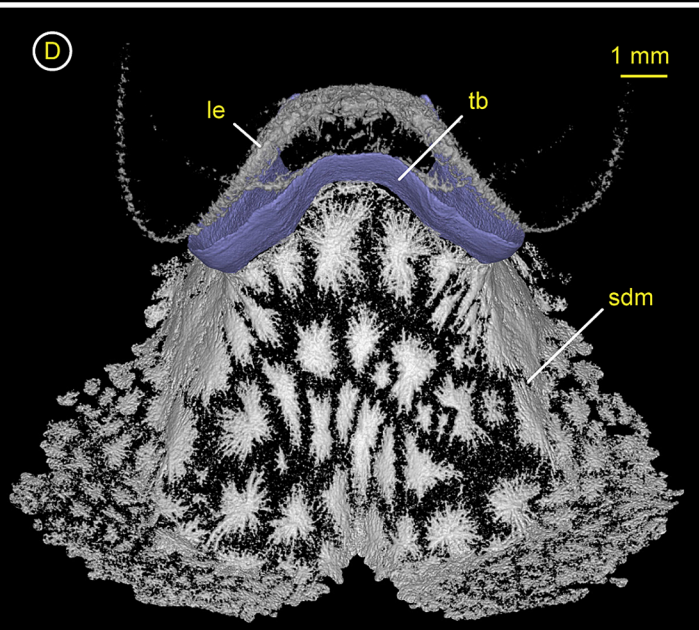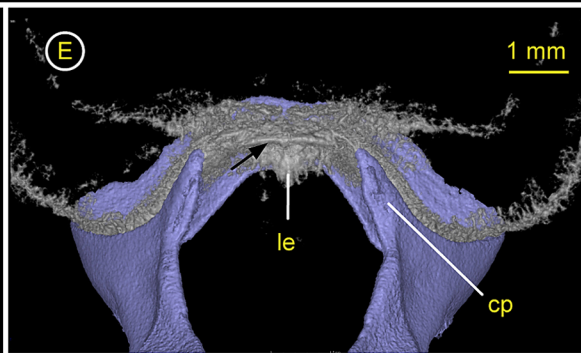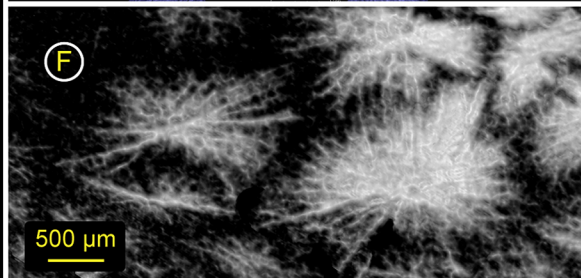

Supplement: Additional file 7: 3D model. — Liothyrella neozelanica. [file 12983_2014_65_MOESM7_ESM.pdf]

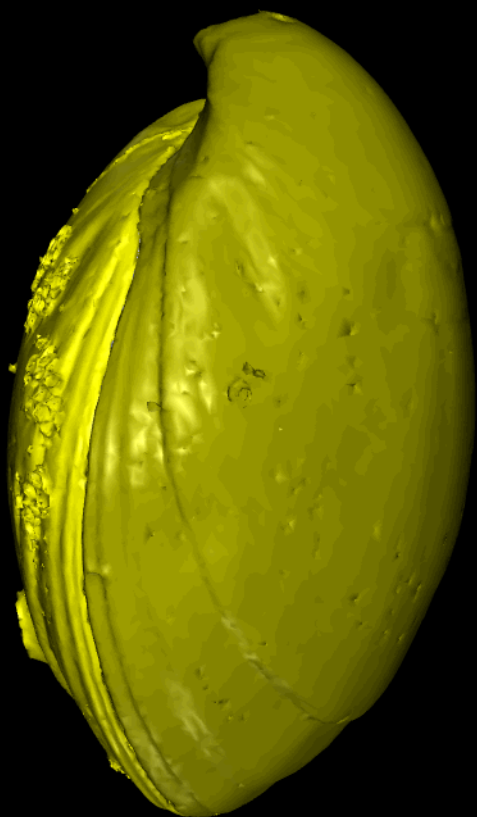

Supplement: Additional file 9: 3D model. — Laqueus rubellus. [file 12983_2014_65_MOESM9_ESM.pdf]

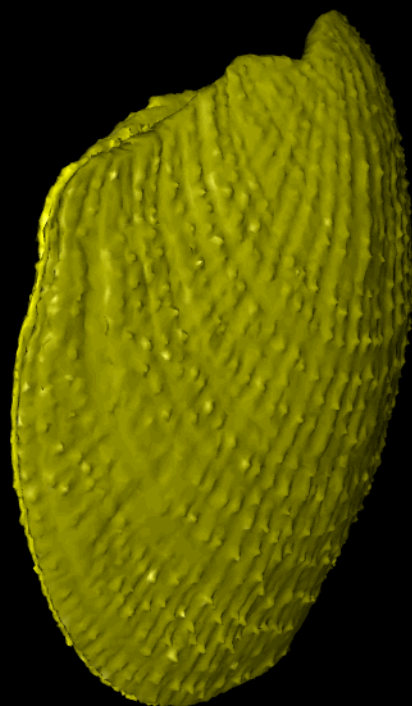

Supplement: Additional file 11: 3D model. — Megerlia truncata. [file 12983_2014_65_MOESM11_ESM.pdf]

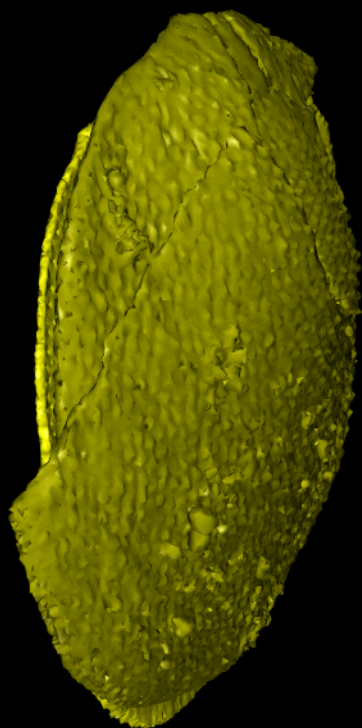

Supplement: Additional file 13: 3D model. — Platidia animioides. [file 12983_2014_65_MOESM13_ESM.pdf]

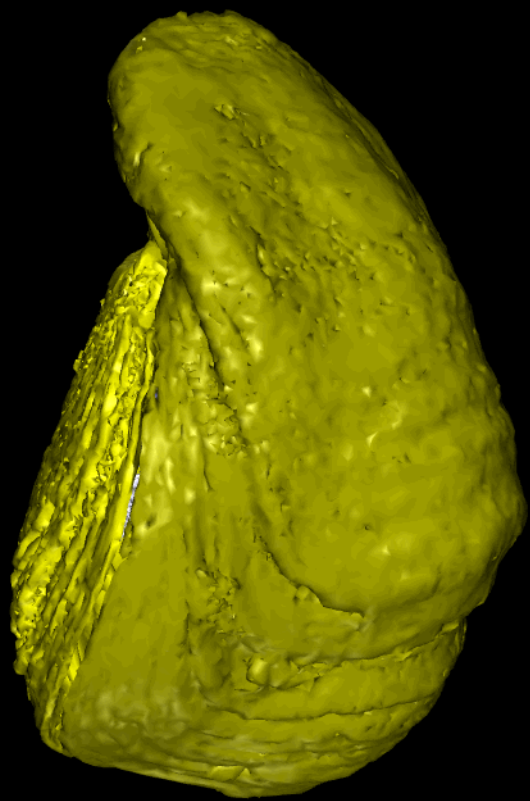

Supplement: Additional file 15: 3D model. — Pumilus aquaticus. [file 12983_2014_65_MOESM15_ESM.pdf]

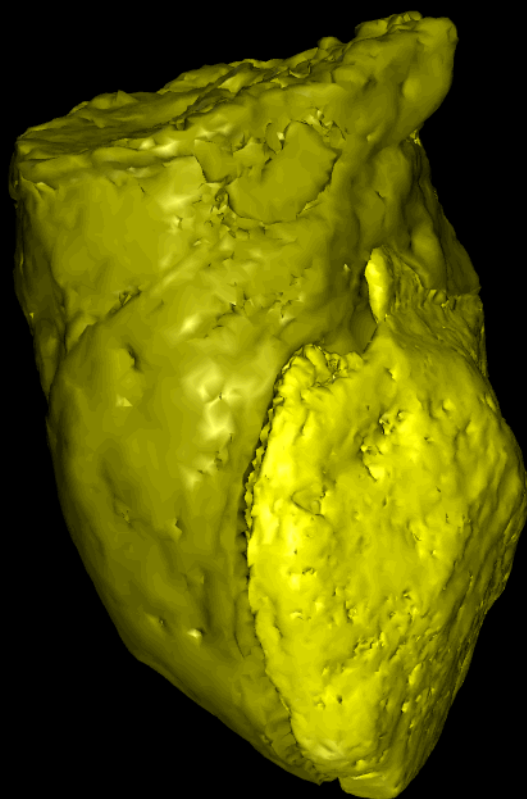

Supplement: Additional file 17: 3D model. — Pajaudina atlantica. [file 12983_2014_65_MOESM17_ESM.pdf]
