# Supplementary material for: Overcoming the fragility – X-ray computed micro-tomography elucidates brachiopod endoskeletons
Source: Front Zool. 2014 Sep 27;11:65. doi: 10.1186/s12983-014-0065-x (PMC4312452; doi:10.1186/s12983-014-0065-x)
Supplement: Additional file 8: Figure S1. — Laqueus rubellus. [file 12983_2014_65_MOESM8_ESM.pdf]

Supplemental Fig. 1 *Laqueus rubellus* (Laqueidae) – ZMB Bra 2259

Ventral valve = top & dorsal valve = bottom, applies to a - f). Scale bars at the top apply to all figures in a column unless indicated otherwise.

a) Anterior view of whole specimen. Shell large, equally convex valves with periodic banding representing changes in the rate of deposition of calcite. Anterior commissure rectimarginate.

b) Lateral view of whole specimen. Both valves equally convex. Dorsal valve shorter than ventral valve. Lateral commissure gently sinuated posteriorly. Beak long, here not incurved, overlying the dorsal umbo.

c) Posterior view of whole specimen, showing lateral dents in both shells (arrows). Small foramen.

d) Anterior view through transparent shell, showing the long looped brachidium (purple) with connection to the median septum **(ms)** of the dorsal valve. Transverse band **(tb)**, transverse plate **(tp)** and spiculation within the body wall (white).

e) Lateral view through transparent shell, showing the brachidium (purple) and spiculation within the upright anterior body wall (white), in front of the loop and beneath the descending lamellae **(dl)**. Faint spiculation within the lophophore (dark gray, arrow), in front of the crural processes, behind the mouth. Ascending lamella **(al)**, descending lamella **(dl)**.

f) Posterior view through transparent shell, showing the strong mantle spiculation near the adductor muscles (arrows). Discontinuity of the very delicate transverse plate is a scanning artefact.

g) Outside of dorsal valve with ventral valve in the background. Whole shell longitudinally oval, straight in front. Dorsal valve oval, longer than wide. Periodic banding increasingly stronger towards the margin, surface punctate. Shell covered with bryozoans, a polychaete tube and small depressions of irregular calcification.

h) Interior of the dorsal valve, with long looped brachidium (purple), mantle and lophophoral spiculation. Hinge line curved, measuring one third of the shell width. Inner and outer hinge plates divided by crural base, inner hinge plate divided along the middle, sockets long and large anteriorly, inner socket ridges narrow, outer socket ridges narrow, slightly elevated and offset from posterior margin. Crus with sharp crural processes pointing anteroventrally. Anterior of crural processes the lophophoral spicules with ribbonlike shape, curved anteriorly. Mantle spiculation on either side of the brachidium (covering the mantle canals) and between the loop forming brachia. Median septum moderately prominent, starting broad at the hinge plate, extending to more than half of the length of the valve. Growth lines visible near the margin. Numerous imprints in the anterior part of the shell, due to irregular calcification.

i) Dorsal view through transparent dorsal valve, showing distribution of spicules of the lophophore (arrow) and mantle. Shell without punctae.

j) Anterior view of brachidium with lophophoral spicules between crural processes, and of dorsal mantle spicules. Mantle spiculation strongest between the brachia, longitudinal arm ranging from crural bases on either side up to anterior brachidium, with two short arms branching off half way towards the margin.

k) Close-up of the dorsal, bilaterally symmetrical mantle spicules between the brachia showing circular arrangement. Single spicules are slender without distinct shape.

l) Posterior view of lophophoral spiculation, showing a ribbon-like structure of the spicules, curved anteriorly, with pointy ends towards the crural processes. Single spicules are slender with no distinct shape and symmetry, and max. 50 µm in diameter.

m) Outside of ventral valve. Shell longitudinally oval, straight in front. As in dorsal valve periodic banding increasingly stronger towards the margin, shell surface punctate.

n) Inside of ventral valve, showing the mantle spiculation. Long, large hinge teeth. Conjoint deltidial plates, line of conjunction visible. Small, and circular foramen, pedicle collar faintly developed.

o) Anterodorsal view of spiculation in the mantle tissue of the ventral valve. The spiculation is strong within the posterolateral body wall and near the hinge teeth where the body wall of ventral and dorsal valve meet, leaving a median hole where the visceral cavity of dorsal and ventral valve are connected. Rather weak spiculation within the anterior body wall, but with a cluster of spicules axially in a dorsoventral orientation. Two mantle canals are covered with spicules laterally on each side. Single spicules are slender and show no distinct shape.

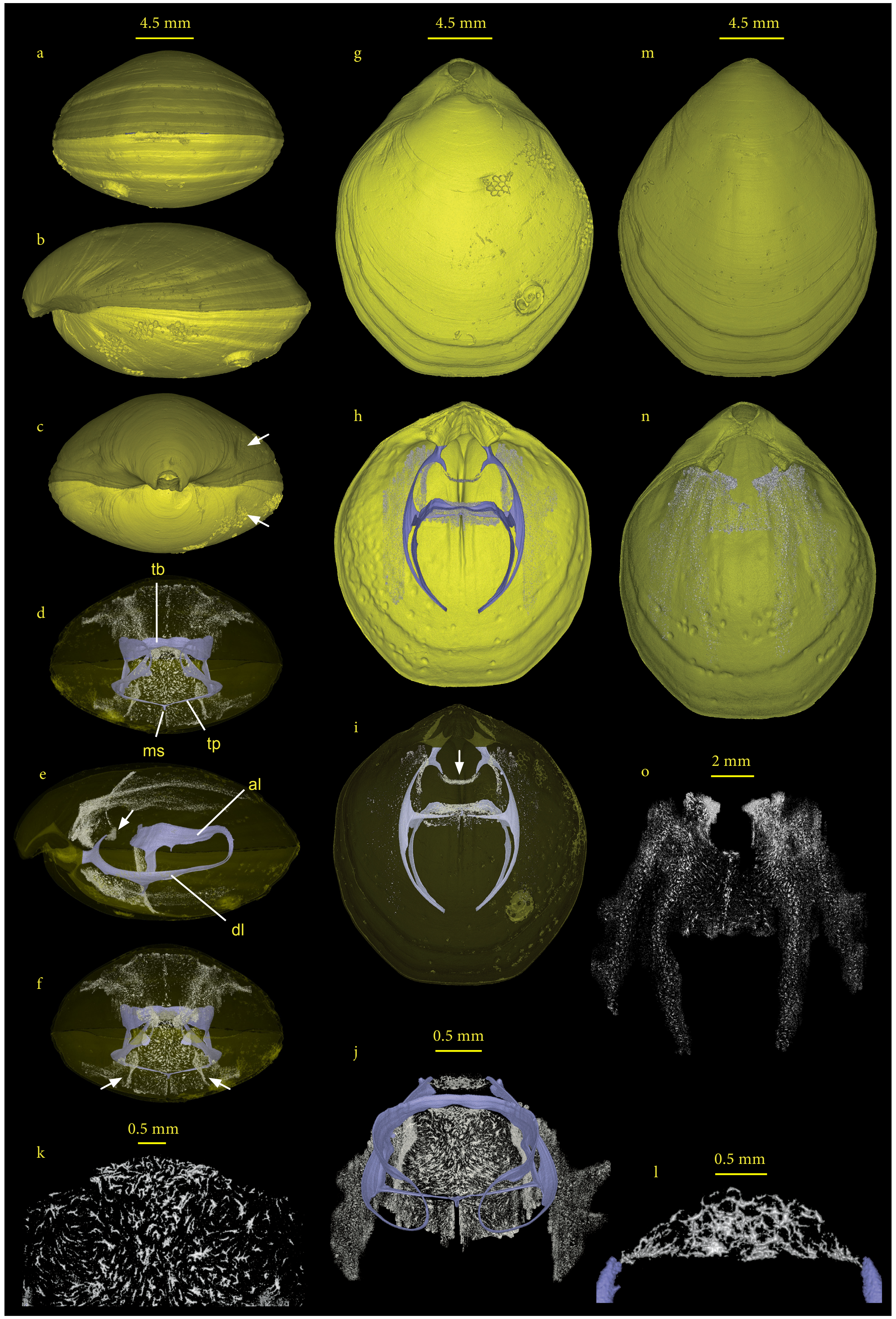

Supplemental Fig. 1 *Laqueus rubellus* (Laqueidae) – ZMB Bra 2259
