# Supplementary material for: Overcoming the fragility – X-ray computed micro-tomography elucidates brachiopod endoskeletons
Source: Front Zool. 2014 Sep 27;11:65. doi: 10.1186/s12983-014-0065-x (PMC4312452; doi:10.1186/s12983-014-0065-x)
Supplement: Additional file 10: Figure S2. — Megerlia truncata. [file 12983_2014_65_MOESM10_ESM.pdf]

Supplemental Fig. 2 *Megerlia truncata* (Kraussinidae) – ZMB Bra 2258

Ventral valve = top & dorsal valve = bottom, applies to a - f). Scale bars at the top apply to all figures in a column unless indicated otherwise.

a) Anterior view of whole specimen. Shell flat, surface of dorsal and ventral valve costate with tubercles and periodic banding. Anterior commissure rectimarginate or unisulcate.

b) Lateral view of whole specimen. Shell biconvex, lateral commissure straight, strongly attrited umbo and dorsal protegulum, ventral valve 1.5 x deeper than dorsal valve.

c) Posterior view of whole specimen, showing large foramen due to shell abrasion. Dorsal and ventral protegulum strongly attrited. Hinge line straight. Dorsal **(di)** and ventral inter area **(vi)**.

d) Anterior view through transparent shell, showing the punctae, the brachidium (purple), the lophophoral endoskeleton **(le)**, and the heavy spiculation within the dorsal mantle **(sdm)** and ventral mantle.

e) Lateral view through transparent shell, showing the brachidium (purple), the spiculation in ventral **(svm)** and dorsal mantle **(sdm)** and in the lateral brachia of the lophophore **(le)**.

f) Posterior view through transparent shell, showing the dorsal **(sdm)** and ventral mantle spiculation **(svm)**.

g) Outside of the dorsal valve with ventral valve in the background. Dorsal valve wider than long. Lateral and anterior margin curved evenly. Surface with punctae and tubercles, tubercles situated on ribs (parvicostellate), pointing towards the margin. Concentric intermitted growth lines, and decrease of concentric rugation towards the anterior margin.

h) Interior of the dorsal valve, showing the brachidium (purple), the relation of the endoskeleton of the plectolophous lophopohore (gray; black arrow = left lateral brachium, white arrow = right spiral brachium) to the dorsal valve, and the spiculation of the body wall. Surface punctate, with big tubercles, margin and border of median septum without tubercles. Median septum arises between the crus, and extends up to the brachidium. Hinge line slightly curved and almost as long as shell width. Inner hinge plates separated widely, anterior and median edges of hinge plates with ridges which fuse and contribute to small crus. Small dental sockets with big accessory sockets, flanked by small outer socket ridges and massive inner socket ridges.

i) Anteroventral view of rich spiculation of the dorsal body wall, inside and laterally from the brachidium. Single spicules plate like, closely spaced to each other, and with interdigitating margins.

j) Close-up of anterodorsal body wall spiculation, showing the plate-shaped structure and the serrated margin of single spicules, and the presence of perforations, margins interdigitating.

k) Outside of the ventral valve. Valve almost as long as wide. Lateral and anterior margin evenly curved. Surface punctate and tubercled, tubercles situated on ribs (parvicostellate) and pointing towards the margin. Concentric and intermitted growth lines, and decreasingly concentric rugation towards the anterior margin.

l) Inside of the ventral valve, showing the spiculation of the body wall. Valve with punctae and few, small tubercles near the margin. Median septum thinner than in dorsal valve, and restricted to the posterior third of the valve. Hinge teeth divided in three elongated knobs, fanlike laterally. Incomplete foramen, pedicle collar well defined, and deltidial plates minute.

m) Anteroventral view of the rich spiculation within the ventral body wall.

n) Close-up of ventral body wall spiculation, showing the plate-shaped structure, the serrated margin of single spicules and the presence of perforations, margins interdigitating.

o) Endoskeleton of plectolophous lophophore with spiral brachium **(sb)** and lateral brachium **(lb)**, and spicules within the proximal part of outer tentacles (arrow). Few loose spicules in front of the mouth (arrowhead).

p) Cross section of left lateral brachium. View from posterior, showing the spiculation of the floor of the food groove and of the base of the dorsal lip **(bl)**.

q) Close-up of the spicules of the lateral brachium, showing the nature of spicules in the dorsal lip and the half-cylinder shaped spicules within the tentacles **(tb)**.

Seidel & Lüter 2014 Supplemental Material

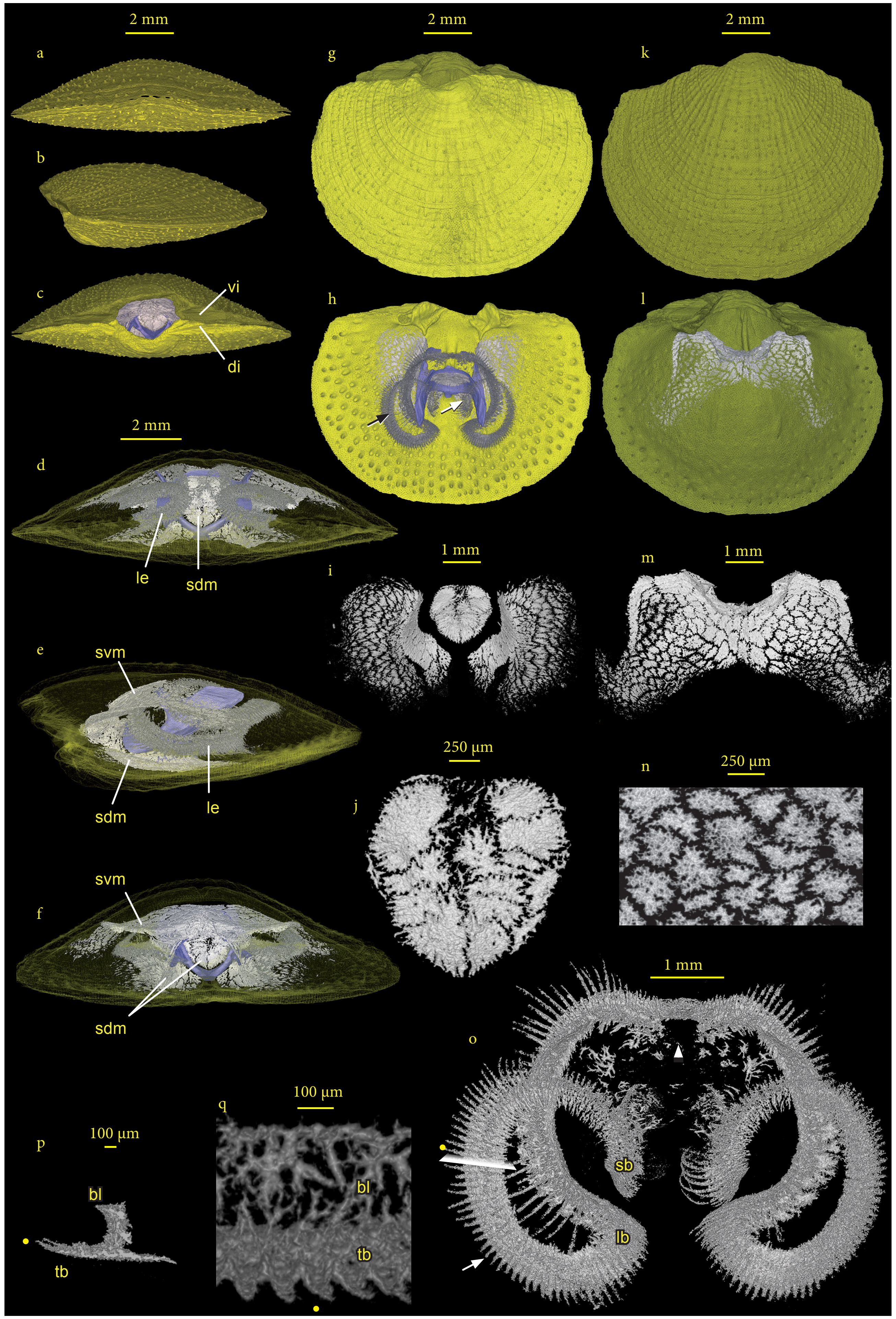

Supplemental Fig. 2 *Megerlia truncata* (Kraussinidae) – ZMB Bra 2258
