# Supplementary material for: Overcoming the fragility – X-ray computed micro-tomography elucidates brachiopod endoskeletons
Source: Front Zool. 2014 Sep 27;11:65. doi: 10.1186/s12983-014-0065-x (PMC4312452; doi:10.1186/s12983-014-0065-x)
Supplement: Additional file 12: Figure S3. — Platidia anomioides. [file 12983_2014_65_MOESM12_ESM.pdf]

Supplemental Fig. 3 *Platidia anomioides* (Platidiidae) – ZMB Bra 2256

Ventral valve = top & dorsal valve = bottom, applies to a - f). Scale bars at the top apply to all figures in a column unless indicated otherwise.

a) Anterior view of whole specimen, ventral valve broken. Shell minute and flat, surface strongly tubercled. Anterior commissure rectimarginate.
b) Lateral view of whole specimen. Dorsal valve less convex than ventral valve. Lateral commissure straight, umbo inconspicuous, and ventral valve 2,7 x deeper than dorsal valve.
c) Posterior view of whole specimen, showing the large foramen in the dorsal Valve. Hinge line straight.
d) Anterior view through transparent shell, showing punctae, brachidium (purple) and spiculation of the lophophore **(le)** and dorsal mantle **(sdm)**.
e) Lateral view through transparent shell, showing the brachidium (purple) with short crural processes **(cp)**, the endoskeleton of the lophophore **(ls)**, and the spiculation within the dorsal body wall **(sdm)**.
f) Posterior view through transparent shell.
g) Interior of the dorsal valve, showing the brachidium (purple), the relation of the endoskeleton of the plectolophous lophophore (gray) to the dorsal valve, and the spiculation of the body wall. Valve circular, slightly wider than long. Large, semi-circle foramen. Inner surface with punctae, and margin prominent. Hinge line straight, dental sockets with faint outer socket ridges, inner socket ridges big, hinge plates minute and widely separated.
h) Outside of the dorsal valve with ventral valve in the background. Surface with faint concentric intermitted growth lines, punctae and with minute tubercles.
i) Outside of the broken ventral valve. Valve as long as wide. Surface punctate, and strongly tubercled in concentric orientation to protegulum.
j) Inside of the ventral valve with minute hinge teeth, notched umbo, curved hinge line, elevated margin, and punctae.
k) Anteroventral view of the spiculation of the dorsal body wall. Spicules long and slender, no distinct orientation, forming an irregular mesh.
l) Close-up of spicules of the dorsal body wall forming an irregular mesh. Single spicules with max. 50 µm in diameter.
m) Ventral view of the endoskeleton of the plectolophous lophophore. Rich spiculation within the s-shaped lophophoral arms at the tentacle bases, and within the proximal part of the tentacles. Centred network of long spicules, separated axially in front of the brachidium, and spreading laterally to form a ventral and dorsal roof within the loops of the two arms (arrow). Posterior spiculation of the lophophoral arms separated between the crural processes, and fused in front of the brachidium. Minor calcification of the right part of the endoskeleton is a scanning artefact.

Seidel & Lüter 2014 Supplemental Material

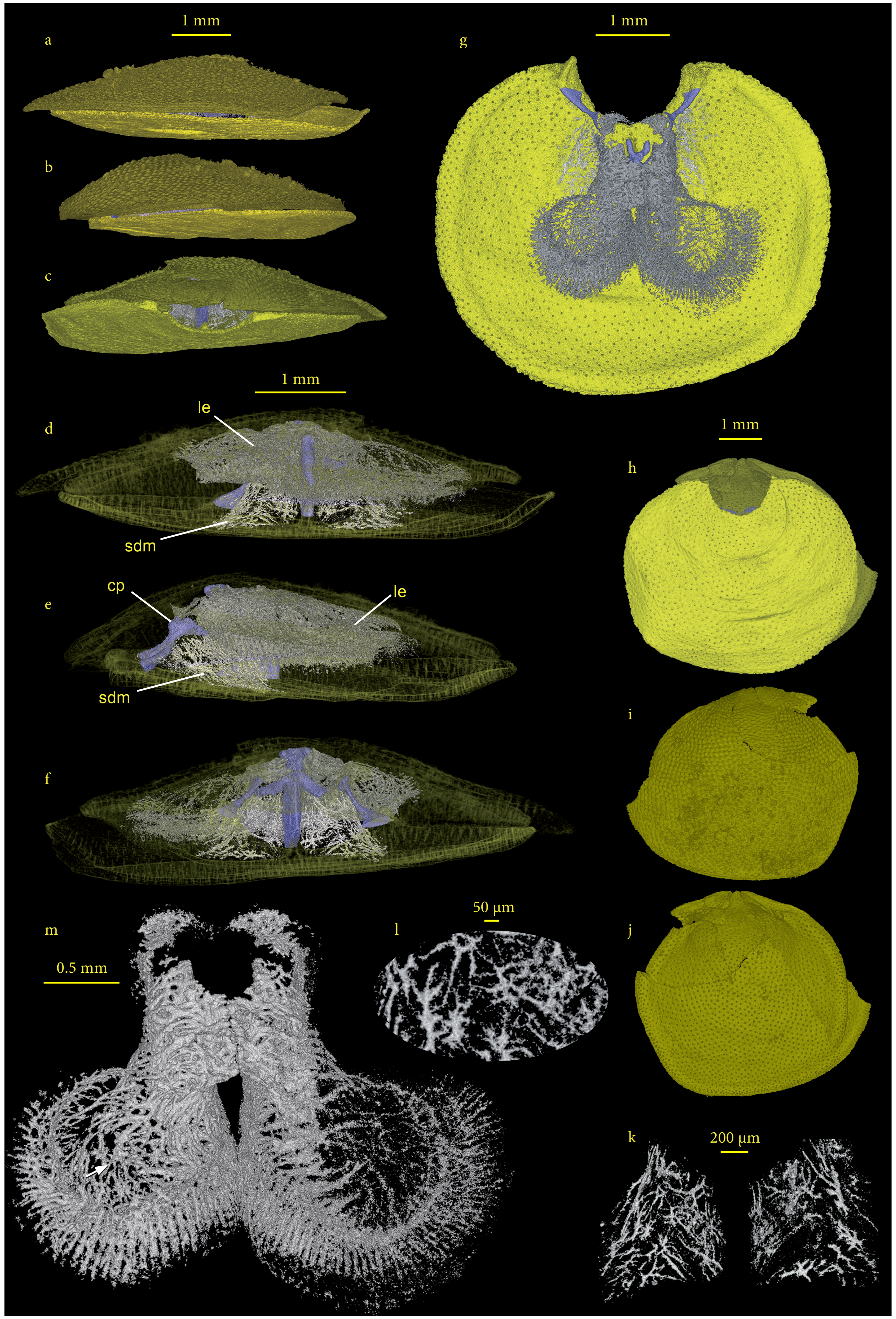

Supplemental Fig. 3 *Platidia anomioides* (Platidiidae) – ZMB Bra 2256
