# Supplementary material for: Overcoming the fragility – X-ray computed micro-tomography elucidates brachiopod endoskeletons
Source: Front Zool. 2014 Sep 27;11:65. doi: 10.1186/s12983-014-0065-x (PMC4312452; doi:10.1186/s12983-014-0065-x)
Supplement: Additional file 14: Figure S4. — Pumilus antiquatus. [file 12983_2014_65_MOESM14_ESM.pdf]

Supplemental Fig. 4 *Pumilus antiquatus* (Kraussinidae) – ZMB Bra 2252

Ventral valve = top & dorsal valve = bottom, applies to a - f) and l - n). Scale bars at the top apply to all figures in a column unless indicated otherwise.

a) Anterior view of whole specimen. Shell thick, and biconvex. Anterior commissure strongly unisulcate. Dorsal valve axially depressed.

b) Lateral view of specimen. Shell biconvex, ventral valve longer than dorsal valve. Recent growth lines strongly marked. Lateral commissure sinusoidal. Beak long, and overlying the dorsal umbo.

c) Posterior view of whole specimen, showing the large and incomplete foramen. Dorsal valve axially depressed.

d) Anterior view through transparent shell, showing punctae and two short lamellae diverging from the median septum (purple), above the endoskeleton of the primitive schizolophous lophophore (gray).

e) Lateral view through transparent shell, showing anterior margin protracted ventrally, punctae and the small median septum with two lamellae (purple), and the small lophophoral endoskeleton compared to the volume of the mantle cavity. The anterior face of the endoskeleton is downgraded, while the lamellae slopes posteriorly, the anterior ends of the endoskeleton sit on the lamellae.

f) Dorsal view through transparent shell, showing the two median lamellae (purple) and the primitive juvenile endoskeleton of the schizolophe.

g) Outside of the dorsal valve with ventral valve in the background. Shell long, elongated. Dorsal valve long, anteroaxially depressed (sulcation), punctate, and recent growth lines strongly marked.

h) Inside of the dorsal valve. Anterior margin protracted ventrally. Submarginal protuberances with elongated ridges towards the articulation, axially two big, and laterally three to four protuberances. Hinge line straight, cardinal process minute, inner socket ridges large projecting over the cardinal border. Low median septum ends anteriorly in two protuberances. Posteriorly from the middle, a median septum with two diverging lamellae forms a short bifurcated support (purple) for the lophophore. Schizolophe endoskeleton consists of two rings supporting each arm, and both rings are positioned laterally of the median septum. No mantle spiculation. Surface punctate.

i) Lophophoral endoskeleton consists of two rings, each with strong spiculation in the anterolaterally part, which continues posteriorly with decreasing density until both rings fuse together axially.

j) Outside of the ventral valve. Valve longitudinal, and outline not triangular but rather shaped like two longitudinally shifted circles, with ventrally elevated anterior margin (sulcation). Growth lines well marked near the margin.

k) Inside of the ventral valve with large, incomplete triangular foramen, pedicle collar well developed. Deltidial plates narrow, hinge teeth small, no dental plates. Very faint median septum in front of the hinge line, and two submarginal tubercles only at the anterior margin. No mantle spiculation. Surface punctate.

l) Anterior view of juvenile whole specimen with minor unisulcation, fine growth lines.

m) Lateral view of whole juvenile shell, ventral valve 1.25x longer than dorsal valve, considerably long beak, not overlying the dorsal umbo, sub-erect. Lateral commissure curved towards the ventral valve.

n) Posterior view of a juvenile whole specimen with large foramen, ventral inter area. No cardinal process.

o) Dorsal view of whole juvenile shell. Shell triangular. Ventral valve in the background, dorsal valve circular, hinge line straight, punctate, concentric growth lines.

p) Inside of the dorsal valve of juvenile specimen. Surface punctate, anteriorly submarginal tubercles, faint median septum in posterior half. Inner socket ridges project behind the straight hinge line. Lophophoral endoskeleton developed anterior and lateral, skeletal arms not fused.

q) Separated skeletal arms of lophophoral endoskeleton. Recent and weakest spiculation at the posterior tips indicating an anterior-posterior gradient in development.

r) Ventral view of ventral valve. Triangular shape, slightly keeled axially, anterior margin evenly curved. Punctate, with stronger recent growth lines.

s) Inside of the ventral valve, incomplete, large and triangular foramen, deltidial plates minute, pedicle collar well developed. Surface punctate.

Seidel & Lüter 2014 Supplemental Material

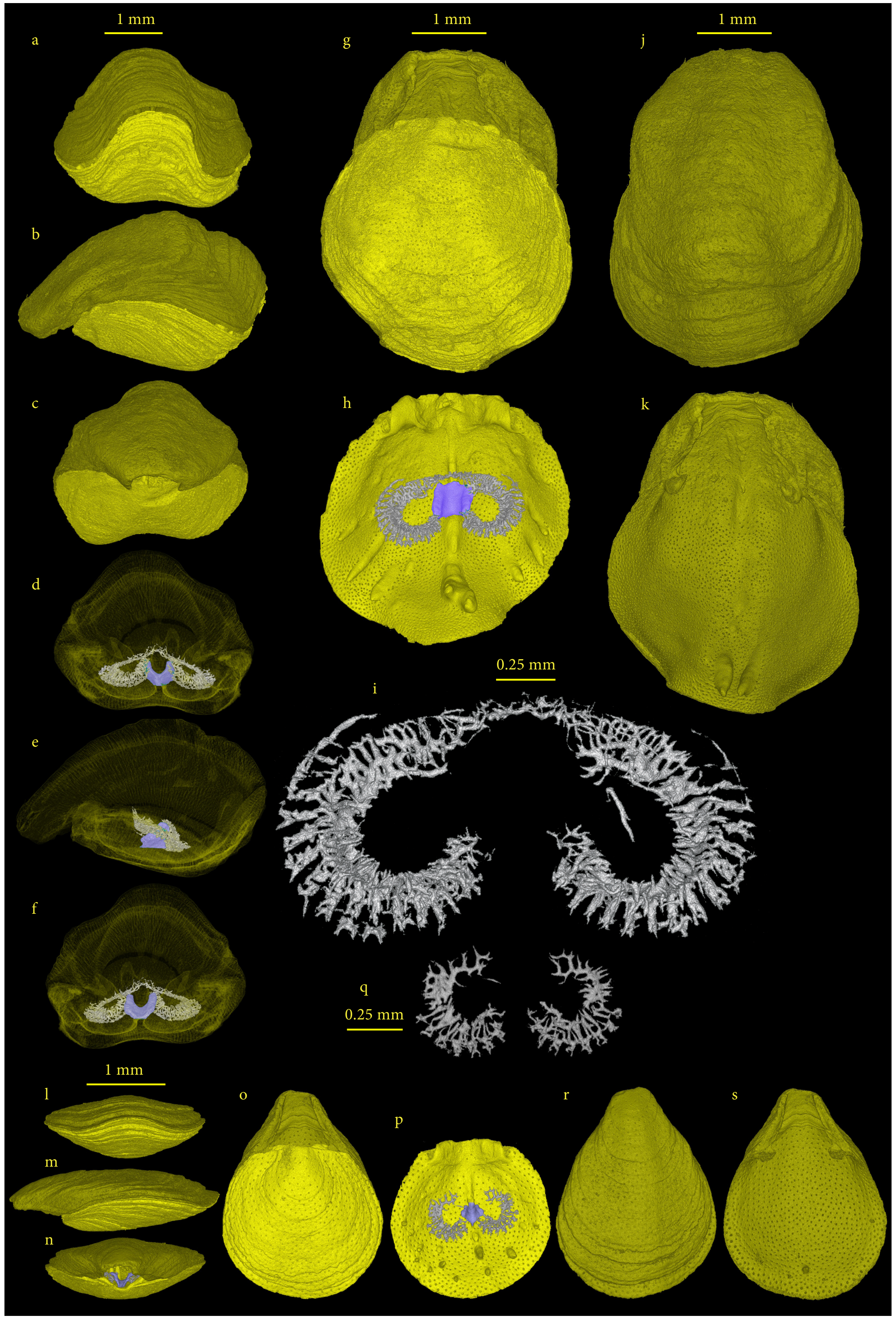

Supplemental Fig. 4 *Pumilus antiquatus* (Kraussinidae) – ZMB Bra 2252
