# Supplementary material for: Overcoming the fragility – X-ray computed micro-tomography elucidates brachiopod endoskeletons
Source: Front Zool. 2014 Sep 27;11:65. doi: 10.1186/s12983-014-0065-x (PMC4312452; doi:10.1186/s12983-014-0065-x)
Supplement: Additional file 16: Figure S5. — Pajaudina atlantica. [file 12983_2014_65_MOESM16_ESM.pdf]

Supplemental Fig. 5 *Pajaudina atlantica* (Thecideidae) – ZMB Bra 2248-2250

ZMB Bra 2248: d, j)
ZMB Bra 2249: a, b, c, e, f)
ZMB Bra 2250: g, h, i, k, l)
Except for h), all images show females.

Dorsal valve = top & ventral valve = bottom, applies to a - d, k). Scale bars at the top apply to all figures in a column unless indicated otherwise.

a) Anterior view of whole specimen. Ventral valve deeper than dorsal valve. Anterior commissure rectimarginate to slightly uniplicate. With attached juvenile (arrow).
b) Lateral view of whole specimen with attached juvenile (arrow). Dorsal valve convex, lid-shaped. Ventral valve large, wedge-shaped, posteriorly with broad attachment side. Lateral commissure straight.
c) Anterior view through transparent ventral valve, showing long punctae canals. Spiculation broken and not shown.
d) Lateral view through transparent ventral valve showing spiculation within the ventral valve.
e) Dorsal view of specimen with ventral valve in the background. Shell large and long. Ventral valve larger, attached, interarea **(ia)** flat, pseudodeltidium **(pd)** triangular and raised. Dorsal valve subcircular, posterior margin straight.
f) Inside the dorsal valve of a female, showing the long cardinal process **(ca)**, and the complex brachidium of the modified ptycholophe lophophore (with two secondary indentions) with major dorsal lobe (mab), minor dorsal lobe (mib), median ramus (mr), ramulus (r). The strongly papillose rim, fuses posteriorly to form the dorsal bridge with marsupial notch (sexual dimorphism only in females) for the passage of specialised, brooding tentacles.
g) Inside the ventral valve showing the spiculation in the body wall and visceral cavity, overlying the lateral gonads. Hinge line straight, teeth strong, bilobate hemyspondylium (hs) projects two prongs anterodorsally, with median myophragm. Pseudodeltidium triangular and raised **(pd)**, interarea **(ia)** flat.
h) Dorsal view through transparent shell of a male, showing the spiculation inside the ventral valve, overlying the lateral gonads.
i) Dorsal view of spiculation within the ventral visceral cavity, see also j)
j) Dorsal view of spiculation within the ventral visceral cavity, comprises two axially separated plates with a mosaic like structure, only connected posteriorly in front of the bilobate hemispondylium, more recent spicules with tree-shape.
k) Transverse section of a female stained with osmium tetroxide to visualise the soft tissue, showing the spiculation within the ventral visceral cavity **(svc)**, late larval developmental stages within the median brood pouch **(la)**, lophophore **(lo)**, digestive diverticula **(dd)** in the dorsal valve and muscles **(mu)**. Dorsal valve **(bv)**, ventral valve **(pv)**, visceral cavity **(vc)**.
l) Transverse section of a female stained with osmium tetroxide, showing the ova **(ov)** and spiculation **(svc)** in the ventral mantle and visceral cavity **(vc)**. Dorsal valve **(bv)**, ventral valve **(pv)**, visceral cavity **(vc)**.

Seidel & Lüter 2014 Supplemental Material

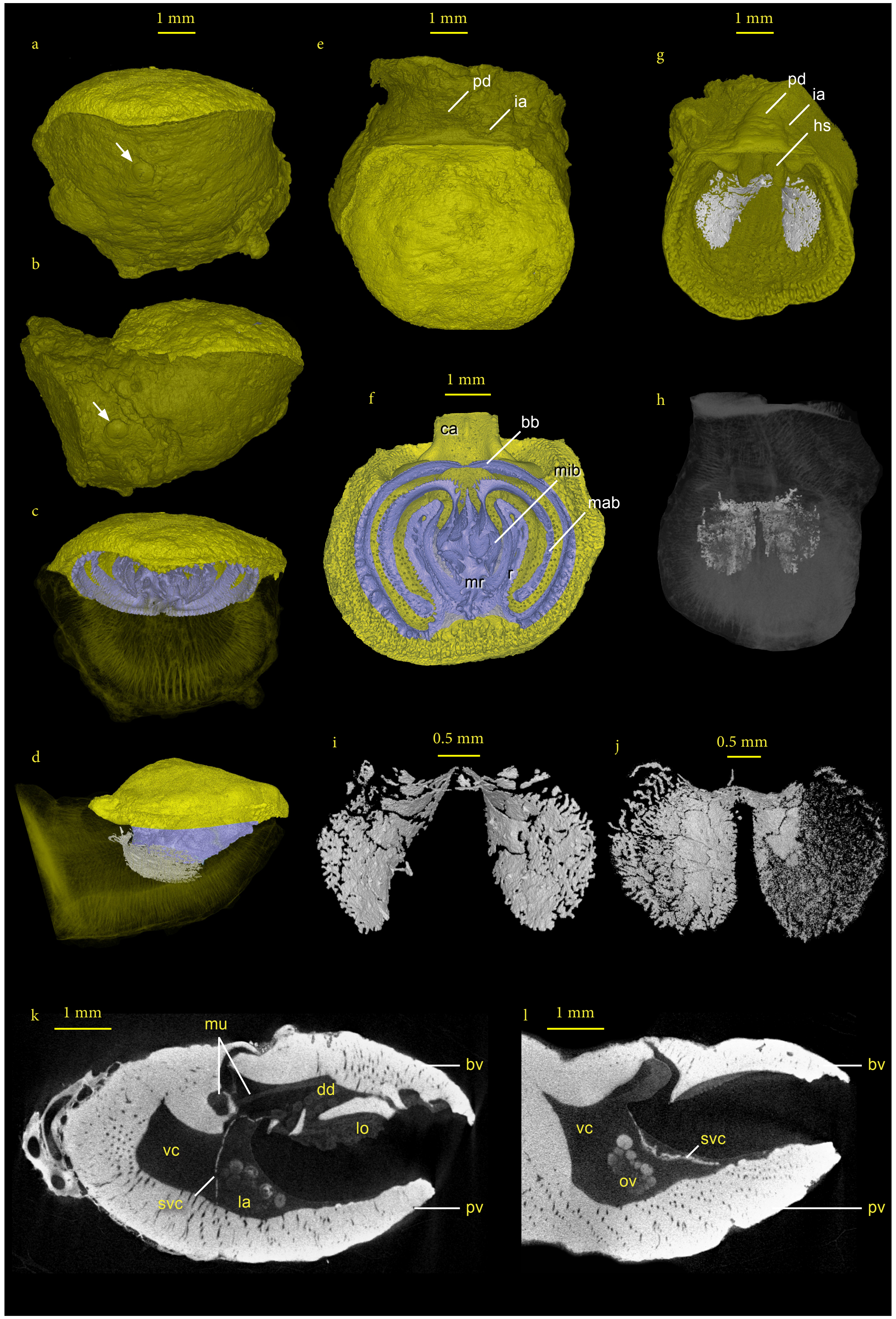


Supplemental Fig. 5 *Pajaudina atlantica* (Thecideidae) – ZMB Bra 2248-2250
