# Supplementary material for: Overcoming the fragility – X-ray computed micro-tomography elucidates brachiopod endoskeletons
Source: Front Zool. 2014 Sep 27;11:65. doi: 10.1186/s12983-014-0065-x (PMC4312452; doi:10.1186/s12983-014-0065-x)
Supplement: Additional file 18: Figure S6. — Phylogeny. [file 12983_2014_65_MOESM18_ESM.pdf]

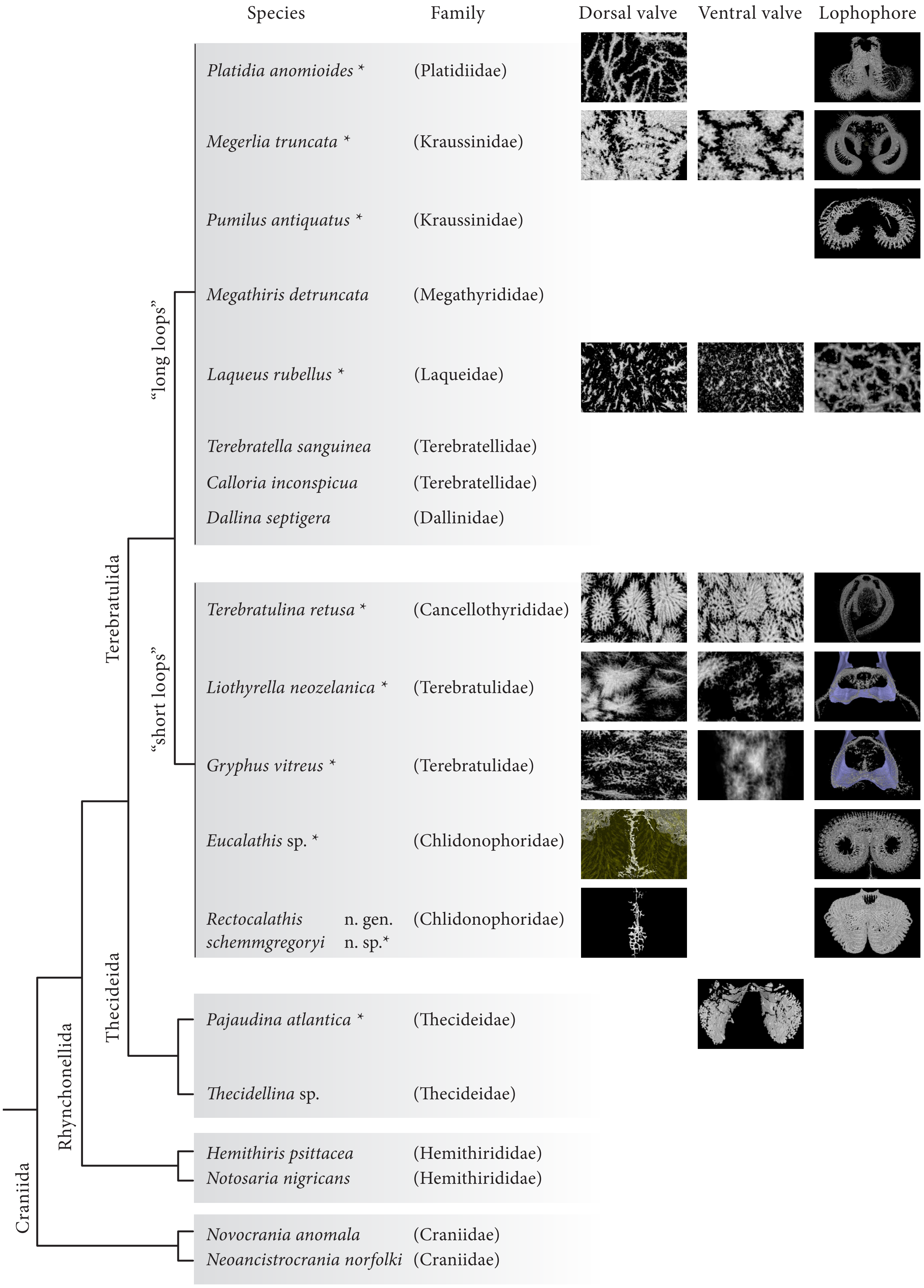

Species
Family
Dorsal valve
Ventral valve
Lophophore
Platidia anomioides *
(Platidiidae)
Megerlia truncata *
(Kraussinidae)
Pumilus antiquatus *
(Kraussinidae)
Megathiris detruncata
(Megathyrididae)
Laqueus rubellus *
(Laqueidae)
Terebratella sanguinea
(Terebratellidae)
Calloria inconspicua
(Terebratellidae)
Dallina septigera
(Dallinidae)
Terebratulina retusa *
(Cancellothyrididae)
Liothyrella neozelanica *
(Terebratulidae)
Gryphus vitreus *
(Terebratulidae)
Eucalathis sp. *
(Chlidonophoridae)
Rectocalathis schemmgregoryi n. gen. n. sp.*
(Chlidonophoridae)
Pajaudina atlantica *
(Thecideidae)
Thecidellina sp.
(Thecideidae)
Hemithiris psittacea
(Hemithirididae)
Notosaria nigricans
(Hemithirididae)
Novocrania anomala
(Craniidae)
Neoancistrocrania norfolki
(Craniidae)
"long loops"
"short loops"
Terebratulida
Thecideida
Rhynchonellida
Craniida
