# Supplementary material for: Overcoming the fragility – X-ray computed micro-tomography elucidates brachiopod endoskeletons
Source: Front Zool. 2014 Sep 27;11:65. doi: 10.1186/s12983-014-0065-x (PMC4312452; doi:10.1186/s12983-014-0065-x)
Supplement: Additional file 19: Table S3. — Scanning parameters of all species of the initial study. Specimens are listed in alphabetical order. P. atlantica 3 was scanned twice using different parameters. [file 12983_2014_65_MOESM19_ESM.pdf]

Specimens	Number Images	Voltage (kV)	Current (µA)	Timing Value (ms)	Averaging & Skip	Magnifi-cation	Scan Time ~ (min)
Calloria inconspicua	900	90	150	750	2 – 1	4,6	45
Dallina septigera	900	90	200	750	2 – 1	3,5	45
Eucalathis sp.	900	60	130	750	2 – 1	13.2	45
Gryphus vitreus	900	80	280	750	2 – 1	3,1	45
Hemithiris psittacea	900	90	200	750	2 – 1	4,4	45
Laqueus rubellus	900	90	200	750	2 – 1	4,4	45
Liothyrella neozelanica	900	80	280	750	2 – 1	2,4	45
Megathiris detruncata	900	60	140	750	2 – 1	12,1	45
Megerlia truncata	900	60	170	750	2 – 1	8	45
Rectocalathis schemmgregoryi	900	60	100	750	2 – 1	17,6	45
Neoancistrocrania norfolki	900	90	150	750	2 – 1	4,9	45
Notosaria nigricans	900	90	150	750	2 – 1	4,9	45
Novocrania anomala	900	60	170	750	2 – 1	8	45
Pajaudina atlantica 1	900	60	170	750	2 – 1	10	45
Pajaudina atlantica 2	900	60	170	750	2 – 1	10	45
Pajaudina atlantica 3	900	35/ 50	350/ 240	750	2 – 1	8	45
Platidia sp.	900	70	110	750	2 – 1	13,3	45
